# Trinucleotide Rolling Circle Amplification: A Novel Method for the Detection of RNA and DNA

**DOI:** 10.3390/mps1020015

**Published:** 2018-04-26

**Authors:** Jean-Marc Zingg, Sylvia Daunert

**Affiliations:** Department of Biochemistry and Molecular Biology, University of Miami, Miami, FL 33136-6129, USA; sdaunert@med.miami.edu

**Keywords:** isothermal amplification, rolling circle amplification, DNA, RNA, detection, lateral flow assay, affinity column, point of care test, diagnostic, Zika virus, Usutu virus, Noro virus, human papilloma virus

## Abstract

Most natural DNA and RNA are devoid of long trinucleotide (TN) sequences that lack one specific nucleotide (missing nucleotide (MN)). Here we developed a novel method that is based on rolling circle amplification (RCA), in which the TN-information of short TN stretches is sequence-specifically recognized, transferred, extended, amplified and detected by padlock probes that consist entirely of nucleotides complementary to the three nucleotides present in the target sequence (complementary TN-information). Upon specific head-to-tail annealing and ligation to the TN-target sequence, these padlock probes represent extended complementary TN versions of the target sequence that can be further amplified by trinucleotide rolling circle amplification (TN-RCA). Since during TN-RCA the MN (as dNTP) is not added, background amplification is minimized with endogenous RNA/DNA (which mostly would require all four dNTP). Therefore, various labelled dNTP can be added to the TN-RCA reaction that enables the separation, isolation and detection of the amplified single-stranded DNA (ssDNA). Here the TN-RCA method is exemplified with RNA/DNA from Zika virus and from human papilloma virus (HPV). TN-RCA is a novel isothermal amplification technique that can be used for sensitive sequence-specific detection and diagnosis of natural and synthetic DNA or RNA containing TN stretches with low background in short time.

## 1. Introduction

Rolling circle amplification (RCA) occurs naturally to replicate the genomes of certain transposons, bacterial plasmids and viruses having circular genomes [1]. Rolling circle amplification has also been developed for a number of in vitro applications, such as for isothermal amplification of circularized DNA templates generating amplification products for sequencing, library construction, bioengineering or diagnosis [2,3,4]. Circularized DNA templates are formed upon connecting the ends of linear DNA by ligases such as T4 ligase from Bacteriophage T4 or *Paramecium bursaria* chlorella virus (PBCV-1) ligase from *Chlorella* virus. For in vitro diagnostics, circular DNA is formed by annealing head-to-tail single-stranded linear 5′-phosphorylated padlock probes of typically 50–200 bp to linear target DNA or RNA and ligase-mediated joining of their ends [5]. Correct non-mismatched annealing is required for most DNA or RNA ligases what has enabled this method to distinguish single point mutations and polymorphisms at the ligation junction [6,7,8,9,10]. In most cases, the circularized padlock probes are extended and amplified by RCA from a start primer or from random hexamer primers (DNA or RNA) that anneal to the circularized padlock probe and initiate the replication reaction by a polymerase with high strand-displacement activity such as Φ29 polymerase or *Bacillus stearothermophilus* (Bst) DNA polymerase [11,12,13]. Whereas RCA is a linear amplification technique, a number of modifications in the RCA technique allow also quasi-exponential amplification, such as the ramification or cascade amplification method (RAM), RCA coupled with loop-mediated amplification (RCA-LAMP), or as recently demonstrated by circle-to-circle amplification (C2CA) RCA [2,3,14,15,16,17,18].

For detection, the linear extended concatemeric single-stranded DNA (ssDNA) amplification products are usually separated as high molecular weight DNA with low migration in matrices such as agarose, polyacrylamide gels or paper, and visualized using fluorescent intercalating dyes such as ethidium bromide or Gel Red [3]. Alternatively, the ssDNA amplification products are detected using a labeled start primer and/or hybridization of labeled oligonucleotide detection probes and molecular beacons (e.g., labeled with dye/gold, fluorescent, biotinylated, digoxigeninated, or radioactive markers). These labels are then detected by optic, colorimetric, enzymatic, or electrochemical signal amplification reactions in vitro as well as in situ, e.g., in paraffin-embedded tissue slides or in fixed cells for localization and diagnosis [19,20,21,22,23,24]. Fluorescent detection has the advantage that multiple RNA/DNA species can be detected simultaneously using spectrally separable dyes [8]. A number of fluorescent-labeled nucleotides have also been tested for direct incorporation during RCA and direct or indirect detection by fluorescence resonance energy transfer (FRET) [25,26]. However, most of these detection methods for RCA products have relatively low sensitivity (e.g., only one or a few labels are usually present in the detection probes), high background due to non-specific random hybridization and to the presence of endogenous high molecular weight DNA, and are time-consuming due to the long time to hybridize and wash the non-specifically bound probes from the sample.

As a diagnostic technique, RCA has been used for detection of DNA or RNA either occurring naturally (genomic or pathogen DNA or RNA) or of synthetic oligonucleotides that have been linked to antibodies (Immuno-RCA) or to microarrays (Surface-RCA) [2]. For Crimean Congo hemorrhagic fever virus, a negative strand RNA virus, complementary DNA (cDNA) was detected by an in situ RCA technique [27], and for human immunodeficiency virus (HIV) cDNA, point mutations were detected using RCA [28]. Recently, a netlike RCA-based point of care test (POCT) was developed for the pH-responsive detection of microRNA [24]. Using the circle-to-circle amplification (C2CA) method [17,18], gold-labeled detection probes and either magnetic beads-based read out or lateral flow on paper strips, drug resistant *Mycobacterium tuberculosis* was detected within 75 min [15,16]. Although a number of studies have shown the ability of RCA to isothermally detect and diagnose pathogens, the lower rate of linear amplification when compared to polymerase chain reaction (PCR) or other exponential amplification techniques (e.g., helicase-dependent amplification (HDA), recombinase polymerase amplification (RPA), loop-mediated isothermal amplification (LAMP)), and the interference of sample-derived background has so far limited the use of RCA-based techniques in any approved diagnostic clinical or laboratory tests.

In this study, we developed a novel RCA technique, trinucleotide RCA (TN-RCA), which is based on 5′-phosphorylated padlock probes (~50–200 bp long) that anneal at low temperature sequence-specifically head-to-tail with their ends to specific target sequences lacking one of the four nucleotides (missing nucleotide (MN)) in DNA or RNA (~20–40 bp TN-stretches). The sequences of such padlock probes completely lack the nucleotide complementary to the MN. Since during TN-RCA the MN (as dNTP) is not added to the reaction mixture during polymerization, only the correctly ligated circular padlock probe will amplify minimizing background amplification that may occur at low temperature in the presence of endogenous RNA or DNA (which mostly would require the presence of all four dNTP). Therefore, in the absence of non-specific amplification, various labeled dNTP can be added to the TN-RCA reaction that enables the separation, isolation and detection of the amplified concatemeric linear ssDNA. In the following, TN-RCA is exemplified with a guanosine-free or G-free padlock probe, and for a specific cytidine-free or C-free target sequence in Zika virus DNA and RNA and for human papilloma virus (HPV) DNA.

## 2. Materials and Methods

### 2.1. Materials

Zika viral particles from Brazilian Fortaleza and Rio strains (kindly provided by M. Ricciardi and Dr. D. Watkins (Department of Pathology, University of Miami, Miami, FL, USA)) were harvested from Vero cells supernatants, filtered using a 0.4 μm filter, and the viral RNA was isolated using an a QIAamp viral RNA minikit (Qiagen, Germantown, MD, USA). For detection of the presence of Zika virus DNA and for generation of Zika DNA target sequences, reverse transcription polymerase chain reaction (RT-PCR) was performed as described below using these samples. All oligonucleotides were purchased from Sigma (Burlington, MA, USA). HeLa cells (ATCC) were grown in DMEM/10% FCS, 2 mmol/L L-glutamine, 100 μg/mL streptomycin and 100 U penicillin and genomic RNA and DNA was isolated with the Trizol method according to the manufacturers’ protocol (Invitrogen, Waltham, MA, USA).

### 2.2. Reverse Transcription Polymerase Chain Reaction

Primer pairs for RT-PCR to detect Zika virus were searched using Primer3 software [29,30]. Alignments of the sequences from Zika virus strains from Uganda (Genbank NC_012532.1), French Polynesia (Genbank KJ776791.1), and Brazil (Genbank KU497555.1) were further used to select primers that would amplify these three viral strains. A suitable primer pair was identified in a sequence coding for the non-structural protein 1 (NS1), with one mismatch towards the Uganda virus strain. No identical sequences were found in human DNA using BlastN searches [31]. Alignments with other Flavivirus sequences (Dengue virus 1 to 4, Genbank KT187564.1, KT187558.1, KR296744.1, KP406806.1), Japanese encephalitis virus (Genbank NC_001437.1), yellow fever virus (Genbank NC_002031.1), and the alpha virus Chikungunya virus (Genbank KJ451624.1 and KJ451623.1) showed that these viruses will not be amplified. RT-PCR was performed in a single tube using the Tth DNA polymerase according to the one-step RT-PCR protocol given by the manufacturer (Roche, Basel, Switzerland). Briefly, 5 μL of viral RNA or particle were assembled in reaction buffer with 1 μL of 50 μM of each primer Zikafw and Zikarv: Zikafw: 5’-GCTTGAAATTCGGTTTGAGG-3’; Zikarv: 5’-CTTTCCTGGGCCTTATCTCC-3’.

The reverse transcription (RT) reaction was at 60 °C for 30 min. Then, the samples were heated to 94 °C for 1 min, followed by 40 cycles at 94 °C, 30 s, at 38 °C, 30 s and at 72 °C, 45 s, with a final elongation step at 72 °C for 7 min. The PCR products were separated by a 2% agarose gel, extracted with a gel extraction kit (Qiagen) and sequenced (Genewiz, South Plainfield, NJ, USA) or used for TN-RCA reactions.

### 2.3. Trinucleotide Rolling Circle Amplification Design

Stretches of 20–30 bp DNA sequences in which cytidine (C) was absent were searched in aligned genomic sequences for Zika virus strains from Uganda (Genbank NC_012532.1), French Polynesia (Genbank KJ776791.1), and Brazil (Genbank KU497555.1). A stretch of 24 bp was identified in a sequence of the nonstructural protein 1 (NS1) partially overlapping with the Zikarv PCR primer, that was C-free and identical in the three viral strains with the exception of one C in the Uganda strain. Interestingly, when the selected target sequence (5’-TGTTGGTATGGAATGGAGATAAGG-3’) was searched using BlastN, 100% homology was found not only with Zika virus, but 95% homology was also found with the Usutu virus, an emerging Flavivirus in Europe [32]. The 5′-phosphorylated G-free padlock probe was so designed that the 5′-end anneals to the first 12 bp of the positive strand of the target, and the 3′-end to the second 12 bp, so that ligation will occur between the two adenosines (A) in the middle. Two A at the target sequence have been reported to be efficiently ligated both by T4 ligase in DNA/DNA [33] as well as by SplintR Ligase in DNA/RNA hybrids [34]. The following oligonucleotides were used as the short synthetic Zika virus target DNA and RNA (bold), and as 5′-phosphorylated Zika virus G-free padlock probes. Zika virus RNA and DNA targets (bold the target sequence): ZTargetupper: 5-TAAAGATGGC**TGTTGGTATGGAATGGAGATAA**GGCCCAGGAAAG-3; ZTargetlower: 5-CTTTCCTGGG**CCTTATCTCCATTCCATACCAACA**GCCATCTTTA-3; ZRNA: 5-UAAAGAUGGC**UGUUGGUAUGGAAUGGAGAUAA**GGCCCAGGAAAG-3.

Padlock probes (bold: ends that anneal to the target sequence; underlined: sequence recognized by the start primer).

Padlock probe a: G-free padlock probe for Zika, GfreeZ (84 bp):

5’-p-**TCCATACCAACA**TTTTTATCTTAACTCACCAACACCATTTTTTCTAATCTCAACCTTACTACACTCTTTTTT**CCTTATCTCCAT**-3’

Padlock probe b: G-free padlock 74, GfreeZ74 (74 bp):

5’-p-**TCCATACCAACA**TTTTTATCTTAACTCACCAACACCATCTCAACCTTACTACACTCTTTTTT**CCTTATCTCCAT**-3’

Padlock probe c: G-free padlock 74 one mismatch, GfreeZ1mis (74 bp, mismatched bases is lower case):

5’-p-**TCCATACCAACA**TTTTTATCTTAACTCACCAACACCATCTCAACCTTACTACACTCTTTTTT**CCcTATCTCCAT**-3

Padlock probe d: G-free padlock 74 two mismatches, GfreeZ2mis (74 bp, mismatched bases are lower case):

5’-p-**TCCATACCAtCA**TTTTTATCTTAACTCACCAACACCATCTCAACCTTACTACACTCTTTTTT**CCcTATCTCCAT**-3’

Start primer A:

5’-TGGTGTTGGTGAGTTAAG-3’

The calculated melting temperature (Tm) of the non-mismatched 5′-end and 3′-ends that anneal to the target sequence were both 34 °C, which is relatively low due to the absence of G in the sequence. The calculated Tm of the start primer was 49 °C. The start primer did not reveal high homology to human DNA in BlastN searches. A search for self-dimers or hairpins in the G-free padlock probe and the start primer using OligoAnalyzer 3.1 (Integrated DNA Technologies, Inc, Coralville, IA, USA) did not reveal any secondary structure that would be disadvantageous.

### 2.4. Considerations for Developing Assay Conditions of TN-RCA for Specific Target Sequences

As described in the following, assay design for TN-RCA may need to take into consideration several specific features that depend on the target type (single/double stranded DNA/RNA, length, target sequence). For efficient TN-RCA with genomic DNA targets, the DNA may be cleaved with restriction endonucleases close to the 3′-side of the padlock probe and then denatured and annealed to the padlock as outlined below. Annealing of DNA/DNA will generate B-DNA type helix of 10 bp per helical turn, which can be ligated efficiently by T4 ligase from Bacteriophage T4 and 10 mM ATP in the presence of fresh dithiothreitol (DTT). The efficiency of ligation has been shown to be dependent on certain nucleotides at the ligation junction that may need to be considered [33]. During amplification by the polymerase cutting the target DNA close to the 3′-side of the padlock probe will facilitate the release of the circular padlock probe from the intertwined genomic DNA and generate a 3′-end for self-priming. Cutting of the target DNA can also be achieved by annealing padlock probes with a T/A mismatch and subsequent cleavage of the adenine in the target sequence by MutY adenine DNA glycosylase. Whole genome amplification and protein nucleic acid (PNA) probes can be used to make the target more accessible for the padlock probes [35].

For efficient TN-RCA with RNA, SplintR Ligase (NEB, Ipswich, MA, USA), PBCV-1 Ligase from *Chlorella* virus, or T4 RNA ligase 2 (Rnl2) is preferentially used [34,36]. Annealing of DNA/RNA will generate A-DNA-like structure having intermediate characteristics between the A- and B-DNA-type structure and between 10 and 11.6 bp per helical turn. During amplification by the polymerase, RNases (e.g., RNase H, RNase A, RNase III) may be added what will hydrolyze specifically the RNA in DNA/RNA hybrids thus releasing the circular padlock probe form the intertwined RNA. When T4 ligase needs to be used with an RNA target, the ATP concentration should be reduced to 10 μM, since otherwise inhibitory AppLigase complexes are formed [33,37].

Most natural DNA and RNA are devoid of long trinucleotide (TN) stretches (20–200 bp) of sequences that lack one specific nucleotide (missing nucleotide (MN)). However, there are exceptions, such as disease-associated trinucleotide expansions, polyAAA tails of mRNA or as present in some pseudogenes. Trinucleotide rolling circle amplification is also expected to work with these special cases, although specific adaptations may be required. For specific applications, dinucleotide RCA (DN-RCA) or even mononucleotide RCA (MN-RCA) can be envisioned, e.g., for the detection in genomic DNA or RNA of dinucleotide repeats or polyAAA tails, respectively, but the suitability of padlock probes containing only two or even one nucleotide for amplification remains to be determined. In these cases, dinucleotide targets can be detected using padlock probes with complementary dinucleotide sequences at the end for annealing, but trinucleotides sequences in the remaining sequence, thus allowing again TN-RCA. In particular, TN-, DN-, or MN-RCA can be used to detect synthetic oligonucleotides that have been linked to antibodies (immuno-RCA) or to microarrays (surface-RCA) [2].

In an optional denaturation/renaturation step prior to the circularization step, the target polynucleotide sequence is denatured (e.g., by heat or alkali for about 5 to 10 min) and then renatured by cooling or neutralization buffer, respectively. Alternatively, the single-stranded target polynucleotide sequence is generated by digesting the strand complementary to the target sequence by using nicking enzymes and exonuclease III [9], or the target DNA is opened by a peptide nucleic acid probe (PNA) [35]. The requirements of this step depend on whether the target polynucleotide sequence is single-stranded or double-stranded DNA or RNA or has secondary structure. High molecular weight polynucleotides (e.g., genomic DNA) can also be physically (e.g., acoustic shearing, ultrasonication, hydrodynamic shear) or enzymatically (e.g., by non-specific nucleases and T7 endonuclease) fragmented, and/or cut by specific restriction endonucleases or nicking endonucleases preferentially at sites close to the target sequence, and/or made single-stranded by whole genome amplification, before denaturation. RNA can also be fragmented chemically by heat and divalent metal cations (magnesium or zinc), or enzymatically by RNase III digestion [38], or RNA is converted to cDNA by reverse transcriptase before denaturation and RNase digestion.

### 2.5. Basic Protocol of TN-RCA Reaction with DNA and G-Free Padlock Probe

5 μL of Zika virus DNA (18 ng/reaction) and 5 μL of HeLa total genomic DNA (120 ng/reaction) as non-specific human background is denatured for 5–10 min with 10 μL lysis/denaturation solution (400 mM KOH, 5 mM EDTA), and then re-natured with 20 μL Neutralization buffer (300 mM Tris-HCl, 200 mM HCl, prepared by mixing 3 mL of 1M Tris-HCl (pH 7.5) and 2 mL of 1 M HCl with 5 mL of water). The pH in the sample after neutralization was between 7.5 and 7.7 as measured using an Amana-1000-L pH Sensor (Innovative Instrument Inc., Tampa, FL, USA). Then, 10 μL of neutralized DNA is immediately added to the ligation reaction mixture containing 3 μL 10× T4 ligase buffer (NEB), 6 μL 1 μM 5′-phosphorylated G-free padlock probe (Sigma), 2 μL T4 DNA Ligase (NEB) (400,000 U/mL), and to 30 μL ultrapure distilled water (Ambion, Austin, TX, USA), and then incubated at 30 °C for 30 min. To this solution a TN-RCA polymerization mixture is added consisting of 6 μL Φ29 10× reaction buffer (NEB), 1.2 μL of 50 μM start primer (Sigma), 0.6 μL of 10 μg/μL BSA (NEB), 1.2 μL dNTP mix (dATP, dGTP, dTTP, 25 mM each or diluted amounts of this stock), labeled Fluorescein-12-dUTP (various amounts of 1 mM stock, ThermoFisher, Waltham, MA, USA), and/or Biotin-11-dUTP (various amounts of 1 mM stock, ThermoFisher), and 1 μL Φ29 Polymerase (NEB) (2000 U/mL), various amounts of ultrapure distilled water (Ambion) to 60 μL, and then incubated at 30 °C for 0.5–6 h. At the end, 10 μL of 6× gel loading dye is added, the sample mixed and separated by 1.5% agarose gel electrophoresis. The gel is photographed under UV light, and the three channels (visible, red, green) visualized using Adobe Photoshop CS 5.1 software (Adobe, San Jose, CA, USA).

### 2.6. Basic Protocol for TN-RCA Reaction with RNA and G-Free Padlock Probe

5 μL of Zika virus RNA synthetic template ZRNA (18 ng/reaction) or Zika virus genomic RNA (40–80 ng/reaction) and 5 μL of HeLa total genomic DNA (120 ng/reaction) is optionally denatured for 5–10 min with 10 μL lysis/denaturation solution (400 mM KOH, 5 mM EDTA), and then re-natured with 20 μL Neutralization buffer (300 mM Tris-HCl, 200 mM HCl, prepared by mixing 3 mL of 1M Tris-HCl (pH 7.5) and 2 mL of 1 M HCl with 5 mL of water) (for ssRNA/ssDNA denaturation/renaturation is optional). Then, 10 μL of neutralized RNA is immediately added to the ligation reaction mixture containing 3 μL 10× Splint Ligase buffer (NEB), 6 μL 1 μM 5′-phosphorylated G-free padlock probe (Sigma), 2 μL SplintR Ligase (NEB) (25,000 U/mL), and to 30 μL ultrapure distilled water (Ambion), and then incubated at 30 °C for 30 min. To this solution a TN-RCA polymerization mixture is added consisting of 6 μL Φ29 10× reaction buffer (NEB), 1.2 μL of 50 μM start primer (Sigma), 0.6 μL of 10 μg/μL bovine serum albumin (BSA; NEB), 1.2 μL dNTP mix (dATP, dGTP, dTTP, 25 mM each or diluted amounts of this stock), labeled Fluorescein-12-dUTP (various amounts of 1 mM stock; ThermoFisher), and/or Biotin-11-dUTP (various amounts of 1 mM stock; ThermoFisher), and 1 μL Φ29 Polymerase (NEB; 2000 U/mL), various amounts of ultrapure distilled water (Ambion) to 60 μL, and then incubated at 30 °C for 0.5–6 h. At the end, 10 μL of 6× gel loading dye is added, the sample mixed and separated by 1.5% agarose gel electrophoresis. The gel is photographed under UV light, and the three channels (visible, red, green) visualized using Adobe Photoshop CS 5.1 software.

### 2.7. Self-Priming TN-RCA in the Absence of Start Primers

For short RNA/DNA targets, the 3′-end of the target oligonucleotide can serve as efficient starting point for self-priming so that no start primer needs to be added. Moreover, since Φ29 polymerase has 3′ to 5′ RNase activity which digests ssRNA [39], the non-digested RNA remaining annealed to the ligated padlock probe can serve as start site of the TN-RCA reaction, so that even for longer target RNA the start primer is not necessary and can be left away in the reaction mixture. For longer RNA targets digestion can be facilitated by adding specific RNases (e.g., RNase III, RNase H) [38,39], whereas longer DNA targets may need to be generated by restriction endonucleases, nickases, whole genome amplification, or dsDNA Fragmentase (NEB). For DNA targets, a self-priming 3′-end can also be generated by annealing padlock probes with a T/A mismatch and subsequent cleavage of the adenine in the target sequence by MutY adenine DNA glycosylase. Accordingly, with DNA, self-priming can be initiated after digesting the non-annealed ssDNA by the 3′ to 5′ exonuclease activity of Φ29 [40].

### 2.8. Generation of Circularized Padlock Probes

Circularized padlock probes were generated using the CircLigase ssDNA Ligase kit according to the manufacturers’ protocol (Epicentre, Madison, WI, USA). Briefly, Padlock probes were incubated for 1 h at 60 °C and CircLigase then inactivated for 10 min at 80 °C.

### 2.9. Digestions with MseI, Exonucleases and RNases

The TN-RCA reaction product was digested with MseI (NEB) (10 U) for 20 min at 37 °C in the presence or absence of cutting primer, Msecutprimer: 5’-TTTATCTTAACTCACCAACT-3’ (underlined: MseI recognition site), and the enzyme subsequently inactivated at 65 °C for 20 min. Lambda exonuclease (NEB) (0.5 U), exonuclease III (NEB) (20 U), exonuclease VIII (NEB) (2 U), T7 exonuclease (2 U), RNase H (NEB) (1 U), RNase A (Thermo Scientific, Waltham, MA, USA) (10 U), RNase A/T1 Mix (Thermo Scientific) (1 U), RNase T1 (Invitrogen/Ambion) (1 U), ShortCut RNase III (NEB) (0.4 U) were added to the TN-RCA reaction mixture after the ligation step was completed, or at various times during the TN-RCA reaction. In some experiments, ShortCut RNase III (NEB) (0.4 U) was also added before the ligation step to fragment the target genomic Zika RNA. Random hexamers (Exo-Resistant Random Primer; 0.2 μL of 500 μM stock; Thermo Scientific) also was added after the ligation step.

### 2.10. Incorporation of dUTP and Digestion with Uracil DNA Glycosylase

1 μL of Deoxy-UTP (dUTP) from various working stock dilutions (1, 0.1, 0.01 mM) (Roche) was added to the TN-RCA reaction mixture, and Uracil-DNA glycosylase (Roche) (1 U) was added at various time points with/without endonuclease IV (NEB) (10 U) as outlined in the Figure legends.

### 2.11. Detection Methods

#### 2.11.1. Agarose Gel 

As described above high molecular weight TN-RCA reaction products are efficiently separated from background signals by 1.5–1.7% agarose gels, and when labeled fluorescein-12-dUTP is added to the reaction, no ethidium bromide or Gel Red is necessary to visualize the products in the gel. Images of agarose gels without ethidium bromide or Gel Red are acquired using the Epi Blue setting by an Azure Biosystems C200 Imaging system (Dublin, CA, USA).

#### 2.11.2. Microtiter Plate

60 μL of the biotinylated TN-RCA reaction is added to the well of a Neutravidin microtiter plate (Pierce Neutravidin Coated High Binding Capacity (HBC) White 96-well Plates with SuperBlock blocking buffer) and incubated for 20 min. The well is washed 3 times with 200 μL PBST (1 tablet PBS (Invitrogen) in 100 mL H_2_O and 100 μL (0.1%) of Tween-20, filtered with a 0.2-µm filter) and blocked with 200 μL PBST/1% BSA for 10 min. Detection is done enzymatically with the Opti-4CN substrate kit (Bio-Rad, Hercules, CA, USA) by adding 150 μL of 1/1000 dilution of Blotting grade avidin-horseradish peroxidase (HRP) in antibody dilution buffer (PBST/1% BSA) for 20 min, washed with 200 μL PBST for 3× 5 min, and detected by adding 0.2 mL of Opti-4CN substrate per 10 mL diluents (mixed one part of Opti-4CN diluent with 9 parts H_2_O). The plate is incubated for up to 30 min with shaking and documented. Alternatively, instead of the microtiter plate, the sample can be dot blotted onto nitrocellulose membranes and air dried and detected as above using the Opti-4CN kit according to the manufacturers’ protocol (Bio-Rad). This method gives no background when the ligase is omitted in the TN-RCA reaction, but it gives a very low background without target DNA/RNA and TN-RCA when the basic protocol is used. This background is most likely due to the extension of the start primer along the linear padlock probe, which leads to incorporation of some biotin-11-dUTP up to the end of the unligated padlock probe (Figure S1). In addition, with synthetic short target RNA/DNA, signals may be generated by annealing the target to padlock probe and extension to the end of the linear padlock probe. As discussed below, background can be avoided by leaving away the start primer and use instead self-priming, e.g., after digestion of the target with various RNases, that digest the non-annealed target generating a free 3′-end for priming.

#### 2.11.3. DNA Affinity Column

Separation using a DNA affinity column is essentially based on the Monarch DNA PCR and DNA cleanup kit (Monarch, NEB) whereas detection is based on the Opti-4CN detection kit (Bio-Rad). 60 μL of the 11-biotin-dUTP labeled TN-RCA reaction is added to 420 μL of binding buffer, then loaded on spin column and centrifuged for 1 min. The column is washed 2× with 200 μL wash buffer and spun for 1 min in between. Then, 200 μL of 1/1000 dilution of blotting grade Avidin-HPR in antibody dilution buffer (PBST/1% BSA) from the Opti-4CN Substrate Kit (Bio-Rad) is added, centrifuged for one minute, washed 2× with 200 μL binding buffer (Monarch, NEB) (spin 1 min in between). Then, one part of Opti-4CN diluent with 9 parts H_2_O is prepared and 0.2 mL of Opti-4CN substrate per 10 mL of diluents is mixed, 200 μL added to the spin column and incubated for 5–30 min. As alternative to the centrifugation step that requires a centrifuge and electricity, the filter is attached to a 10 mL syringe, and the same procedure is performed by manually generating a vacuum and sucking all the solutions into the syringe.

#### 2.11.4. Lateral Flow Assay

12-Fluorescein-dUTP- and 11-Biotin-dUTP- labeled samples (1–10 μL of TN-RCA reaction) were spotted on the Hybridetect Dipstick, processed as described in the manufacturers’ protocol (Milenia Biotec, Giessen, Germany), and photographed. Alternatively, 12-Fluorescein-dUTP labeled samples (1–10 μL of TN-RCA reaction) were spotted on the Hybridetect Dipstick, processed as described in the manufacturers protocol (Milenia Biotec) but with the addition of 1 μL of 50 μM 5′-biotin-labeled detection probe able to hybridize to the TN-RCA product (Biotin-5’-CTCAACCTTACTACACTC-3’) to the running buffer, and photographed. The band intensity was assessed using the AlphaEaseFC Software (AlphaInotech, San Leandro, CA, USA).

### 2.12. In Situ TN-RCA

The oligonucleotide containing the Zika virus target sequence (bold) (ZTargetamine: 5’-amine-C6-MMT-TAAAGATGGCT**GTTGGTATGGAATGGAGATAAGG**CCCAGGAAAG-3’) was covalently attached to an IgG secondary antibody (goat anti-rabbit IgG, whole molecule; Sigma), using an oligonucleotide-conjugation kit according to the manufacturers’ procedure (Abcam, Cambridge, MA, USA). HeLa cells were grown in Falcon 8-well cell culture slides, fixed with 10% formalin/PBS, permeabilized with saponin (0.05%) and incubated with primary (anti-Akt1/2/3 (Santa Cruz, Dallas, TX, USA)) and oligonucleotide-labeled secondary antibody using standard protocols as for immunofluorescence staining, and TN-RCA performed in situ essentially as described above, washed with PBS, and photographed with a fluorescent microscope (BZ-X710, Keyence, Itasca, IL, USA).

## 3. Results

### 3.1. Design of TN-RCA

**Figure 1:**
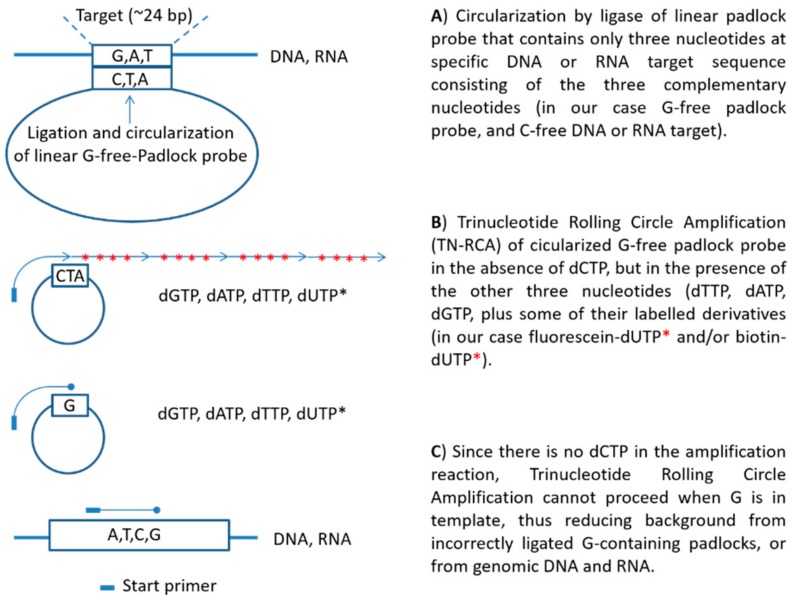
Principle of trinucleotide rolling circle amplification (TN-RCA).

A scheme depicting the principle of the TN-RCA method is shown in Figure 1. When compared to RCA, the ends of the padlock probe oligonucleotides in the TN-RCA method target specifically DNA or RNA stretches in which one or more nucleotides are missing (missing nucleotide, MN), thus facilitating correct annealing and ligation at low temperature. In addition, the base complementary to the missing base in the target sequence is completely absent in the TN-RCA padlock probe, so that upon ligation, the circular template used for amplification consists only of three nucleotides. In the amplification reaction, the missing nucleotide is left away so that only correctly ligated circular templates can be amplified reducing background from mispriming and genomic DNA or RNA or from contaminating DNA/RNA in enzyme preparations [41]. Moreover, this allows incorporating labeled dNTPs (such as fluorescein-12-dUTP, biotin-11-dUTP, digoxigenin-dUTP, radiolabeled dNTP, ethynyl-dNTP [42], bromo-dUTP (BrdUTP), etc.) for later capture and/or optical, fluorescent, bioluminescent, chemiluminescent or enzymatic detection. dUTP is efficiently incorporated by the Φ29 polymerase, which has only a 2-fold lower efficiency of incorporation when compared to dTTP [43]. Depending on the enzymes and labels used, all reaction steps can occur at a constant temperature currently set between 20–40 °C, overall assay time is estimated to be within 0.5–4 h, depending on the steps used, the concentration of the target DNA/RNA and the equipment available (e.g., by using microfluidics or automated microtiter plates the time between the steps can be shortened).

### 3.2. Optimization of TN-RCA

**Figure 2:**
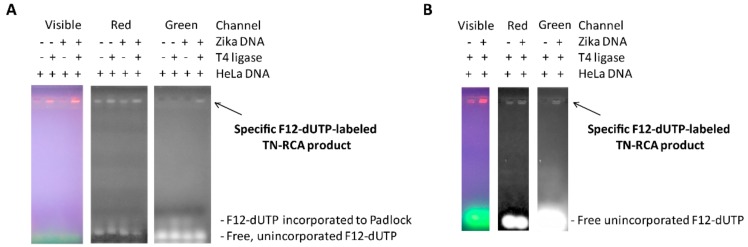
TN-RCA with G-free padlock probe and Zika virus DNA is dependent on the presence of T4 DNA Ligase. (**A**) TN-RCA with G-free padlock probe, denaturation/renaturation 5 min, ligation 30 min, TN-RCA in the absence of dCTP and in the presence of fluorescein-12-dUTP (F12-dUTP); per lane 10 μM for 6 h, agarose gel 1 h, all reactions isothermally at 30 °C. Note the presence of specific amplification in the presence of Zika virus DNA (18 ng/lane) and T4 DNA ligase, the absence of specific amplification in the absence of T4 DNA ligase when observed in the green channel, and the presence of non-specific background signals due to genomic HeLa DNA (120 ng/lane) when observed in the other channels. Note also the presence of low molecular weight DNA signals in the bottom of the gel, coming from F12-dUTP incorporated to start primer extending along the linear padlock probe; (**B**) TN-RCA as in (**A**) but for 1 h and with more fluorescein-12-dUTP (per lane 83 μM).

Since Zika virus RNA at the time of development was of limited availability, the TN-RCA method is exemplified and tested first using Zika virus DNA. A Zika PCR DNA fragment containing the TN target sequence was generated, spiked or not spiked into HeLa cell genomic DNA as non-specific human background, denatured/renatured, and TN-RCA performed with and without T4 DNA ligase and in the presence of 420 μM dNTP (without dCTP) and 16 μM fluorescein-12-dUTP as outlined in the methods section. After separation of the TN-RCA products in a 1.5% agarose gel containing low amounts of Gel Red (Figure 2A), a stronger signal was detected after UV illumination in lanes representing TN-RCA product and background genomic DNA. When observed in the red channel, signals were observed in all lanes. However, when observed in the green channel, only the specifically fluorescently labeled TN-RCA product derived from amplification of Zika virus DNA was detected.

Amplification for shorter times (1h) with increased amounts of fluorescein-dUTP (5× or 80 μM) still resulted in a TN-RCA product specifically detectable only in the green channel (Figure 2B), albeit 16 μM dNTP was found to be already limiting (not shown), so that in the following experiments 80 μM dNTP (without dCTP) was used. Lowering dNTP was possible also because almost none are used for other reactions (K_m_ and K_d_ of Φ29 for unmodified dNTP are 0.5 μM [44] and 1.4 μM, respectively [45]). In the complete absence of dTTP even more fluorescein-12-dUTP may be incorporated; however, amplification to high molecular weight TN-RCA products appears to be less efficient and quenching of fluorescence may occur when too dense [46]. For consistency, all these and subsequent experiments were performed at 30 °C; however, TN-RCA tested at ambient temperature (22 °C) was fully functional albeit slightly less efficient as detected in agarose gels. Since detection of fluorescent TN-RCA products do not require additional detection by fluorescent dyes in the agarose gel (Gel Red, ethidium bromide), subsequent experiments were separated by agarose gels in the absence of these dyes.

### 3.3. Detection of Amplified TN-RCA Products

**Figure 3:**
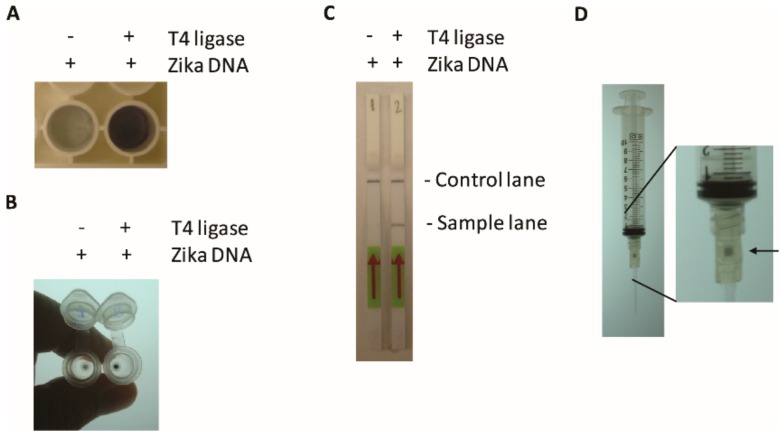
Methods of detection of TN-RCA products in addition to agarose gel electrophoresis (as shown in Figure 2); (**A**) Detection of biotinylated TN-RCA products in Neutravidin-coated microtiter plates using Avidin-HRP and the Opti-4CN substrate for color development; (**B**) Detection of biotinylated TN-RCA products with DNA affinity column using avidin-horseradish peroxidase (HRP) and Opti-4CN substrate for color development; (**C**) Detection of biotin- and fluorescein-labelled TN-RCA products by lateral flow assay using dipsticks and gold-coupled anti-fluorescein antibodies for visual detection; (**D**) syringe with DNA affinity column and detection as in (**B**) (see materials and methods for more details, only methods with visual detection are shown).

TN-RCA generates a labeled high molecular weight TN-RCA extension product and a 1.5% agarose gel efficiently separates it from labeled free nucleotides and shorter background extension products. However, agarose gel electrophoresis requires some equipment, time and electricity and since it is without enzymatic signal amplification it is of relatively low sensitivity. Therefore, alternative detection methods were assessed that may be useful for detection, e.g., for applications on site in a point of care setting. The TN-RCA reaction was performed with DNA and RNA targets using biotin-11-dUTP and added to either a Neutravidin-coated microtiter plate and after washing detected using an enzymatic reaction with horseradish peroxidase (HRP)-linked avidin (Figure 3A). Alternatively, the TN-RCA reaction products were added to a DNA affinity column, centrifuged, washed and again detected using HRP-linked avidin (Figure 3B). A third method of detection is based on a lateral flow assay with dipsticks that capture the biotinylated TN-RCA amplification products and detect it with gold-coupled anti-fluorescein antibodies (Figure 3C). Detection with a DNA affinity column can also be done manually by generating a vacuum using a syringe (Figure 3D), as outlined in the methods section. Although there are more sensitive enzymatic methods available suitable for detection of the labelled TN-RCA product, since they all would also need sensitive readers mostly not available on site in a point of care setting, in this study only detection methods were tested that generate visible products that do not need any further equipment for reading.

### 3.4. Sensitivity of the TN-RCA Assay with the Basic Protocol

**Figure 4:**
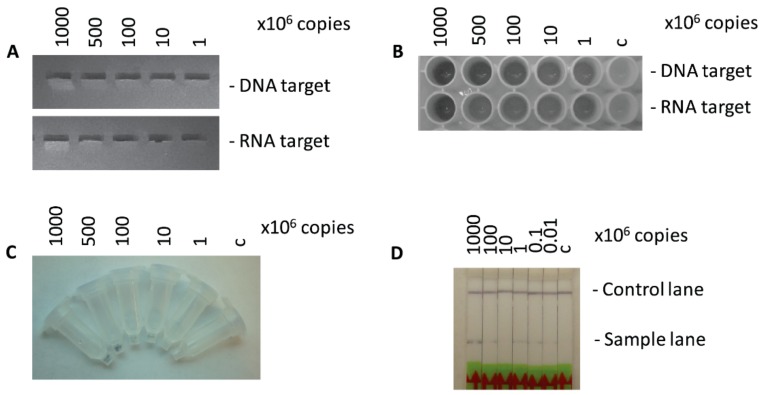
Detection limits of serial dilutions of synthetic Zika DNA/RNA with TN-RCA. (**A**) Agarose gel electrophoresis; (**B**) microtiter plate, Neutravidin-coated; (**C**) DNA affinity column with dilutions of Zika virus RNA; (**D**) Lateral flow assay with dipsticks with dilutions of Zika virus RNA. Detection as in Figure 3 with standard conditions (30 min ligation, 2 h amplification, visual detection).

To determine the limits of detection of TN-RCA using the basic conditions outlined in the methods section (30 °C, 30 min ligation, 2 h TN-RCA) and the various visual detection methods, Zika DNA and RNA templates was serially diluted and TN-RCA performed and detected either by microtiter plates, by DNA affinity columns or lateral flow on paper dipsticks. Depending on the detection method, the detection limits were determined to be in the range of 10^5^–10^6^ copies of input DNA or RNA (Figure 4), what is in the range of Zika virus reported to be present in patient samples in the acute phase (urine, blood) (10^2^–10^6^ PFU/mL) [47], and is comparable to the pM detection limit of other RCA-based visual detection methods [23]. To reach a lower detection limit, the time of amplification can be lengthened or the sensitivity of detection can be increased by adding a step to the basic protocol (see later Figures S5–S9), by enzymatic signal amplification and sensitive equipment/readers not available in a point of care setting. At the detection limit, the signal was still clearly visible, but at the limit of being distinguishable from samples done in the absence of target DNA or RNA, whereas the background in the absence of added ligase or polymerase was negligible. Therefore, some of this signals in the absence of target DNA must come from short labeled extensions from the start primer or the target sequence to the end of the linear padlock probe (Figure 2A and Figure S1) which are dsDNA or dsRNA/DNA when compared to the concatemeric long ssDNA extensions from rolling circle amplification. Thus, it was tested whether these background signals are susceptible to enzymes that specifically digest double-stranded DNA/DNA or RNA/DNA hybrids such as exonuclease III, and some reduction was observed (Figure S1) [12,48,49].

### 3.5. Optimization of TN-RCA for Genomic Zika Virus RNA

**Figure 5:**
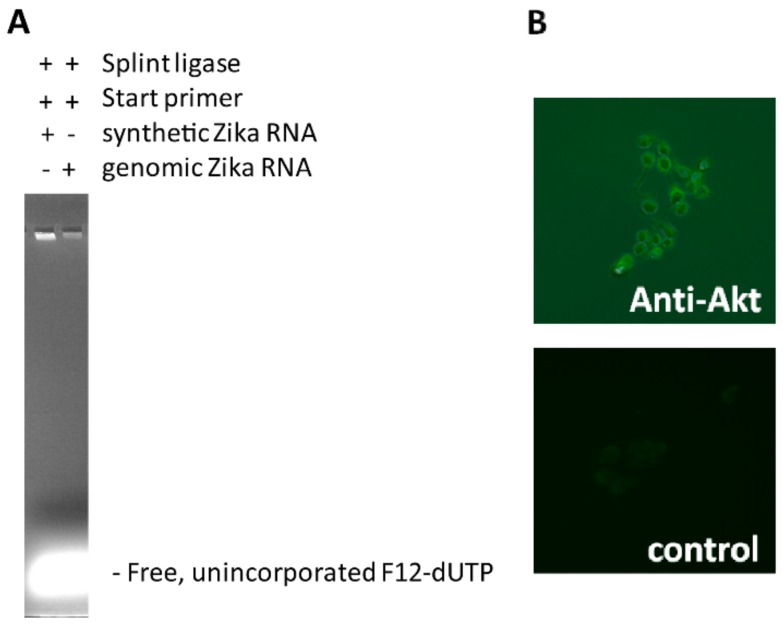
Detection of Zika virus genomic RNA and in situ TN-RCA with oligonucleotide-labeled secondary antibody. TN-RCA with (**A**) Zika virus synthetic and genomic RNA; (**B**) In situ TN-RCA in HeLa cells using rabbit anti-human Akt as primary antibody, IgG covalently labeled with target oligonucleotide as secondary antibody, and TN-RCA as detection method.

To determine the ability to detect intact Zika virus RNA, Zika virus genomic RNA was isolated from supernatants of Zika-virus infected Vero cells. When compared to short synthetic Zika virus target RNA, using Zika virus genomic RNA gave less efficient TN-RCA amplification (Figure 5A), even when amounts and conditions were varied. Interestingly, when exonucleases were added, a robust amplification was observed even with genomic Zika virus RNA (Figure S2). However, further studies using exonucleases revealed that for unknown reasons the increase in TN-RCA amplification was associated with increased self-ligation of the padlock probe in the absence of target DNA/RNA so that overall exonucleases were associated with a decreased specificity. In contrast, short target oligonucleotides that have been covalently attached to antibodies efficiently amplified by self-priming and were detectable by fluorescence microscopy (Figure 5B); thus, similar to the RCA method, which allowed the localization and detection of single target molecules using fluorescence microscopy [35], TN-RCA may be useful for detection of specific cellular targets such as DNA/RNA directly or attached to proteins/antibodies, possibly with lower background [4]. TN-RCA also worked with other target sequences such as human papilloma virus (HPV) DNA and Noro virus RNA (Figure S3).

**Figure 6:**
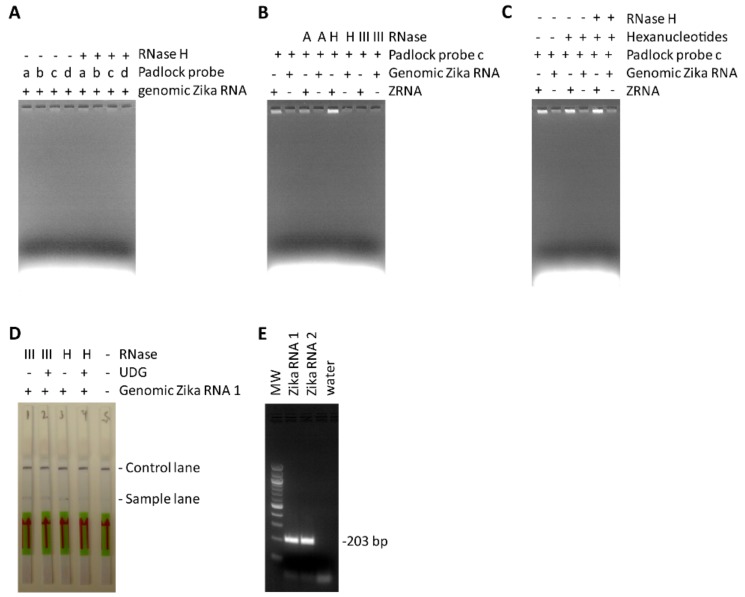
Improving the reaction conditions of TN-RCA with genomic Zika RNA. (**A**–**C**) The reaction conditions and protocol for TN-RCA has been modified to allow more sensitive and specific detection of genomic DNA and RNA. (**A**) Variations of padlock probes (a) 84 bp, (b) 74 bp, (c) 74 bp with one mismatch, (d) 74 with 2 mismatches, in the presence and absence of RNase H; (**B**) variations of RNases with Padlock probe (c); (**C**) variations of random hexanucleotides and RNase H with padlock probe (c); (**D**) lateral flow assay with dipstick with TN-RCA reactions with genomic Zika RNA and improved reaction conditions containing random hexanucleotides, dUTP, UDG, and 12-fluorescein-dUTP and 11-biotin-dUTP, total assay time 3 h; (**E**) RT-PCR with genomic Zika RNA (samples 1 and 2), 40 cycles for 2.5 h plus 1 h separation on agarose gel, total 3.5 h.

Based on these results it was concluded that the best strategy to reduce background and improve TN-RCA is to leave away the start primer and instead use self-priming by the target DNA/RNA to initiate the amplification reaction [50]. Whereas self-priming is easily achieved with short synthetic DNA/RNA targets, longer genomic RNA has to be fragmented to generate a free 3′ end. The intrinsic 3′ to 5′ RNase activity of Φ29 polymerase can digest the RNA that is not hybridized to the target sequence, generating 3′-ends that can be used as starting point for self-priming in the absence of start primer [39]. The digestion of RNA by Φ29 is facilitated by RNase III which cleaves dsRNA often formed in longer RNA targets [38]. Therefore, to improve the reaction conditions for genomic Zika RNA targets, a set of available RNases (RNase H, RNase A, RNase III) was tested to cleave the target RNA before or during the TN-RCA reaction so that self-priming in the absence of the start primer can occur more efficiently (Figure 6A,B). Moreover, conditions of TN-RCA were further optimized by changing padlock probe length from originally 84 bp to 74 bp (equivalent to about 6 helical turns of B-DNA), and by using padlock probes that anneal to their target with one or two mismatches (e.g., to facilitate melting and cleavage by RNases) (Figure 6A,B). It can be assumed that the presence of a mismatch and the addition of an RNase not only specifically hydrolyzed the dsRNA and the RNA in RNA/DNA hybrids thus facilitating self-priming, but also released the circular padlock probe from the intertwined RNA [38].

### 3.6. Development of Two-Step TN-RCA

To facilitate detection with lateral flow assay and to generate templates able to serve as targets in a second amplification step, several methods were tested to fragment the fluorescein- and/or biotin-labeled TN-RCA reaction products after or during the assay. In these experiments, circularized padlock probes (cLPadlocks) were used to evaluate whether the fragments generated in a first TN-RCA amplification can serve as targets and primers for a second TN-RCA amplification. It was found that digestion with a restriction enzyme (MseI) was feasible but required the addition of the complementary G-free oligonucleotide containing the restriction site (Figure S4A); the addition of a circularized padlock probe did only weakly increase amplification suggesting that the digested partially double-stranded fragments did not efficiently serve as target for a second TN-RCA reaction (Figure S4B). Digestion by a restriction enzyme and religation of the generated fragments is used in the circle-2-circle amplification (C2CA) method, but it requires several steps including enzyme cutting, inactivation by heating, religation, and second RCA that would not be practical for a point of care detection method [17,18].

It was also tested whether the incorporation of fluorescein-12-dUTP makes the TN-RCA reaction product sensitive to digestion by uracil DNA glycosylase (UDG) and endonuclease IV, which was not the case (Figure S5A). Therefore, dUTP was added to the TN-RCA reaction mixture at various concentrations and time points in the presence and absence of UDG and endonuclease IV and the reaction products separated by agarose gel (Figure S5B). The UDG alone efficiently fragmented the TN-RCA reaction products most likely since the abasic sites are quite unstable, what is in particular the case in the presence of DTT, NaCl, EDTA, and BSA, components that are in the ligation and amplification assay [51]. The presence of endonuclease IV did not enhance much fragmentation by UDG when compared to UDG alone, although it is reported to have considerable activity with ssDNA [52]. Unexpectedly, in the presence of circularized padlock probes, the presence of endonuclease IV gave strong background signals assumed to be the result of generation of non-specific starting points for Φ29 [53], so that in subsequent experiments endonuclease IV was not used.

The uracil DNA glycosylase fragmented reaction products from the first unlabeled TN-RCA reaction did not serve as efficient targets for a second complete ligation/amplification TN-RCA reaction, possibly because ligation is not efficient with targets containing dUTP and abasic sites after UDG digestion (Figure S6A,B); however, the addition of a circularized padlock probe to the reaction increased TN-RCA amplification with UDG-digested TN-RCA suggesting that the UDG-digested fragments can serve as targets and enable self-priming (Figure S6A,C). Secondary TN-RCA amplification was increased by the addition of circularized padlock probes to UDG-digested TN-RCA products from genomic Zika RNA, but less with products from ZRNA most likely since the signal band was already maximal (Figure S6D,E), altogether suggesting that the fragments generated in the presence of dUTP and UDG could serve as primers for a secondary TN-RCA reaction.

Instead of two-step TN-RCA, the generation of labelled products can also be increased by adding exo-resistant random hexamer oligonucleotides which anneal to the TN-RCA reaction product and incorporate F12-dUTP during the TN-RCA reaction (Figure 6C). However, since random hexamers and the products generated by random hexamers would anneal to pre-circularized padlock probes and efficiently start TN-RCA, only un-ligated complementary G- and C-free padlock probes can be envisioned in a secondary amplification step, albeit at least with UDG-digested TN-RCA products these targets were not enabling efficient ligation in a second TN-RCA reaction (Figure S6A,B). However, the addition of random hexamer oligonucleotides and RNase H to the reaction mixture appeared to increase the efficiency of TN-RCA by facilitating priming and incorporating more labels, and genomic Zika virus RNA could be detected by TN-RCA and lateral flow assay with dipsticks in about the same time (3h) as with conventional RT-PCR and agarose gel (Figure 6D,E).

### 3.7. Sensitivity of the TN-RCA Assay with the Two-Step TN-RCA Protocol

The basic TN-RCA protocol with dipstick detection gave a limit of detection of 10^5^–10^6^ copies (Figure 4D), although the signal bands were generally weak and towards the limit of detection difficult to distinguish from background. To determine the influence of background RNA/DNA, synthetic Zika RNA (ZRNA) was spiked into HeLa RNA, HeLa genomic DNA, and denatured/renatured HeLa genomic DNA, and amplified using either basic TN-RCA (dNTP without dCTP) or RCA (four dNTP) conditions. Using the same padlock consisting of only three nucleotides allows comparing the TN-RCA with the RCA assay conditions, although a padlock consisting of four nucleotides as normally used for RCA is expected to increase background even more since at low temperature it may anneal and ligate at more target sequences. Interestingly, not much background amplification was detected (Figure S7A,B) with the exception of the denatured/renatured HeLa genomic DNA sample and with RCA conditions (Figure S7C), suggesting that with the basic TN-RCA/RCA protocols (30 min ligation, 2 h amplification, visual detection) the appearance of background with RCA depends on the properties of the input background DNA/RNA. Much more background was observed in particular for the RCA conditions when using the two-step protocol with ZRNA spiked into larger amounts of HeLa genomic RNA (Figure S8A,B), and with genomic Zika RNA spiked into HeLa genomic RNA with extended amplification time (Figure S8C,D).

To determine the limit of detection using the two-step protocol in the presence and absence of background RNA, ZRNA was serially diluted and amplified with the two-step protocol. In the absence of HeLa RNA, the detection limit was still in the range of 10^5^ copies as with the basic TN-RCA protocol; however, the signals were generally stronger and easily distinguishable from background (Figure S9A). When ZRNA was spiked into HeLa genomic RNA, experimental variations were higher and the signals were more difficult to distinguish from background amplification (Figure S9B). These results suggest that for a diagnostic application the two-step TN-RCA assay conditions (quality/purity/enrichment of the sample, timing, amounts, and concentrations) may need to be carefully optimized, in particular when low amounts of targets should be detected.

To compare the TN-RCA with the RCA method, a conventional RCA padlock was designed and compared to the TN-RCA padlock. In general, conventional RCA generated more background in particular in the presence of HeLa DNA (Figure S10A) and more so with denatured HeLa DNA (Figure S10B,C). Towards the limit of detection, it was more difficult to distinguish the signal from background for RCA when compared to TN-RCA (Figure S11A,B). This can be explained by incorporation of labelled dNTPs into accessible 3′-ends generated by the normal padlock and start primer and present in denatured HeLa genomic DNA (what is the basis of the whole genome amplification method [54]) and subsequent detection as background.

## 4. Discussion

**Figure 7:**
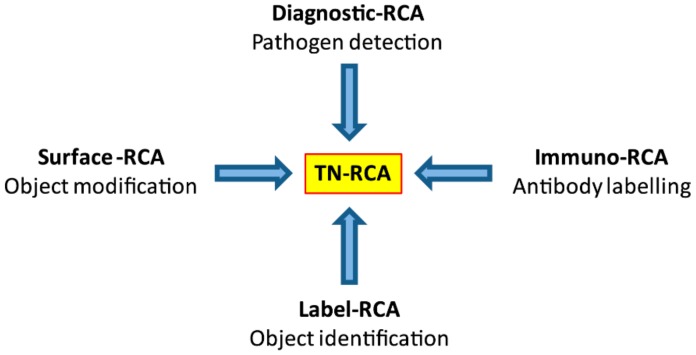
Applications of TN-RCA and advantages over RCA. The trinucleotide rolling circle amplification has several advantages in applications used for conventional RCA, in particular when used at low temperature: lower background since internal DNA/RNA is not amplified; increased and direct specific labelling; and less secondary structure preventing self-annealing. Possible applications of TN-RCA are the diagnosis and detection of pathogens (bacteria, viruses, parasites) in situ or in test tubes/microfluidics (diagnostic-TN-RCA), labelling and detection of proteins (e.g., antibodies) (immuno-TN-RCA), identification (label-TN-RCA) and modification (surface-TN-RCA) of objects and surfaces.

Trinucleotide rolling circle amplification is a novel isothermal amplification technique that can be used for the sensitive detection and diagnosis of any natural or synthetic DNA or RNA containing short stretches of sequences with only three of the four nucleotides with low background in short time (Figure 7). Since all the reactions and detection can be performed in liquid form at low temperature, it can be envisioned that TN-RCA can work also on a microfluidics or automated microtiter-based platform, e.g., for large-scale screening of donated blood samples. Similar to RCA, TN-RCA can also be used for in situ detection, semi-quantitative assessment and localization of DNA or RNA (and of specific point mutations) in tissue sections, including frozen and paraffin-embedded tissue sections, in fixed cells, as well as in dried samples, e.g., of dried urine, saliva, blood or in other forensic samples [9]. For commercial applications, TN-RCA can be envisioned as point of care test (POCT), as clinical, laboratory or field kits or as laboratory technique for in vitro and in situ measurement of specific DNA or RNA. Moreover, TN-RCA can be used to label, detect and identify/authenticate other molecules (e.g., antibodies, proteins, lipids, nucleic acids, organisms/genetically-modified organisms (GMOs), chemicals, solutions such as color in paintings or biometric ink [55] in writings or microdots [56]), and other objects of interest that have been tagged or spiked with oligonucleotides (e.g., stabilized with phosphothionate linkage) encoding the complementary target sequence. As reviewed by Ali et al. for RCA [3], TN-RCA can also be envisioned for numerous applications in nanobiotechnology and materials sciences (e.g., as template for nanoassemblies and nanostructures such as nanowires, nanoribbons and nanotubes, DNA-based metamaterials for drug delivery, hydrogels, DNA glues), with the advantage of lower self-aggregation of the TN-RCA products and lower background due to less formation of multimeric by-products as result of self-annealing of the padlock probes and start primer.

As outlined in the following and exemplified in this study, TN-RCA has several characteristics that may be advantageous over conventional RCA in particular when used at low temperature. First of all, TN-RCA has increased specificity of amplification and lower background. Due to the presence of only three dNTPs (in our case dCTP is missing) in the reaction, only correctly ligated circular G-free padlock probes will amplify and incorporate labeled dNTPs into the TN-RCA product what increases the specific signal and lowers the signals from background amplification from endogenous DNA/RNA. In cases utilizing the presence of a gap between the ends of the padlock, that is either filled in by an enzyme or an oligonucleotide [7], TN-RCA may have advantages as well since incorrect filled-in ligations that incorporate a G into the padlock probe will not amplify. High molecular weight DNA/RNA and their complexes with proteins that often co-migrate with the RCA product and give background will not be detected (Figure 2), since fluorescein-12-dUTP is only incorporated after specific amplification into TN-RCA products. Moreover, since all polymerase molecules and dNTPs are used only for specific amplification, they are not consumed in non-specific incorporations, thus enhancing the specific incorporation at lower concentrations. In RCA, background signals can be generated from the presence of high molecular weight genomic DNA in the sample/enzyme preparations [41], from amplification due to presence of nicks in genomic DNA or RNA (what is the basis of whole-genome amplification [54]), or from non-specific annealing and/or amplification of padlock probe, start primer, or labelled detection probe to genomic DNA or RNA. The generation of background signals also depends on the method of detection and on the properties and purity of the sample. Labelled dNTPs have been used in conventional RCA [25] and RCA for in situ detection [19], but in complex samples they may be incorporated non-specifically giving background, whereas in TN-RCA this does not occur. Optionally, as in RCA, labeled start primers (e.g., with fluorescein, biotin, digoxigenin) can be used for additional labeling, attachment or isolation of the TN-RCA products what may enable also multiplexing and/or automation e.g., using microtiter plates.

A second advantage of TN-RCA is the potentially increased sensitivity and shorter assay time. For detection, when compared using labeled detection probes that usually contain one label per probe and thus also one label per detected concatemeric repeat in the RCA product, TN-RCA can incorporate many and multiple-type labeled dNTP (e.g., the G-free padlock probe used has 22 adenines to incorporate labeled dUTP). Since the labeled dNTPs are incorporated during the TN-RCA, lengthy hybridization procedures can be avoided as they occur for example with microtiter and in situ assays.

A third advantage of TN-RCA is increased specificity at lower reaction temperatures. Due to the absence of guanosine in the G-free padlock probe, or alternatively due to the absence of cytidine in the C-free padlock probe, which are both nucleotides forming Watson–Crick-type triple-bonds with high affinity in DNA and RNA, the overall melting temperature is lower with consequent lower secondary structure of padlock probe and target sequence as well as lower self-priming and self-annealing features of the padlock probe. Similarly, the start primer has a lower secondary structure and self-dimer and self-priming features again reducing background. These features allow to anneal the ends of the padlock probes at lower temperature (e.g., in our case isothermally at 20–37 °C) and/or the usage of longer padlock probe ends for annealing to the target sequences, what leads to a higher specificity and sensitivity. Moreover, since stretches of 20–30 bp or longer target sequences lacking a particular nucleotide are rare in the genome (due to the overall equal presence of all four nucleotides and their random and coding-biased distribution), the specificity of TN-RCA is higher, although it requires the presence of such TN stretches in the target sequences. This requirement can be overcome by the incorporation of “universal base analogues” into the end-sequences of the padlock sequence, in particular when the trinucleotide-target sequence contains one or a few of the forth otherwise missing base to facilitate annealing [57], but still only three dNTP are required for TN-RCA amplification, thus increasing the number of potential target sequences that are accessible to TN-RCA. The identification of unique TN stretches in natural DNA can be facilitated by developing software able to screen genomic databases such as Genbank [58].

A disadvantage of TN-RCA is the limit of being able to target only stretches of DNA/RNA containing three or less nucleotides. This may limit the versatility of the method with certain targets (e.g., for shorter genomes of viruses or specific genes). Since after sodium bisulfite treatment of DNA/RNA only cytosine and not 5-methylcytosine is converted to uracil [59,60], TN-RCA may also be a method to distinguish un-methylated over methylated cytosines in specific target sequences. Taken together, by targeting only stretches of DNA/RNA containing three or less nucleotides, the TN-RCA method gains features that increase specificity, sensitivity, and lowers background. Similar to the RCA method, variations of the TN-RCA steps and assay conditions can be imagined that increase these features even further.

## 5. Patents

Patent application for TN-RCA (PCT/US17/50292).

## Supplementary Materials

The following are available online at http://www.mdpi.com/2409-9279/1/2/15/s1. Figure S1: Background reduction of TN-RCA, Figure S2: Optimization of TN-RCA with exonucleases, Figure S3: Detection of human papilloma virus (HPV) DNA and Noro virus RNA, Figure S4: Development of two-step TN-RCA, Figure S5: Development of two-step TN-RCA, Figure S6: Development of two-step TN-RCA, Figure S7: Influence of background RNA or DNA on TN-RCA and RCA with basic protocol, Figure S8: Influence of background RNA on TN-RCA and RCA with two-step protocol, Figure S9: Limit of detection in the presence of background RNA with two step TN-RCA protocol, Figure S10: Comparison of methods TN-RCA and RCA in the presence of background DNA, Figure S11: Limit of detection with conventional RCA and TN-RCA using the basic protocol.
