# Supplementary material for: Trinucleotide Rolling Circle Amplification: A Novel Method for the Detection of RNA and DNA
Source: Methods Protoc. 2018 Apr 26;1(2):15. doi: 10.3390/mps1020015 (PMC6526412; doi:10.3390/mps1020015)
Supplement: Supplementary file 1 [file mps-01-00015-s001.pdf]

methods and protocols

MDPI

Benchmark

Supplemental Data File with Figures S1–S11 Trinucleotide Rolling Circle Amplification: A Novel Method for the Detection of RNA and DNA

Jean-Marc Zingg * and Sylvia Daunert

Department of Biochemistry and Molecular Biology, University of Miami, Miami, FL 33136-6129, USA
* Correspondence: jaz42@miami.edu; Tel.: +1-305-243-3531

1. Background reduction with exonucleases

Possible reasons for background include the synthesis of short labeled fragments from the start primer and the unligated padlock probe or signals derived from the self-primed DNA/RNA target (**Figure S1**). As outlined, since these fragments are double-stranded they may be susceptible to digestion with RNases or exonucleases.

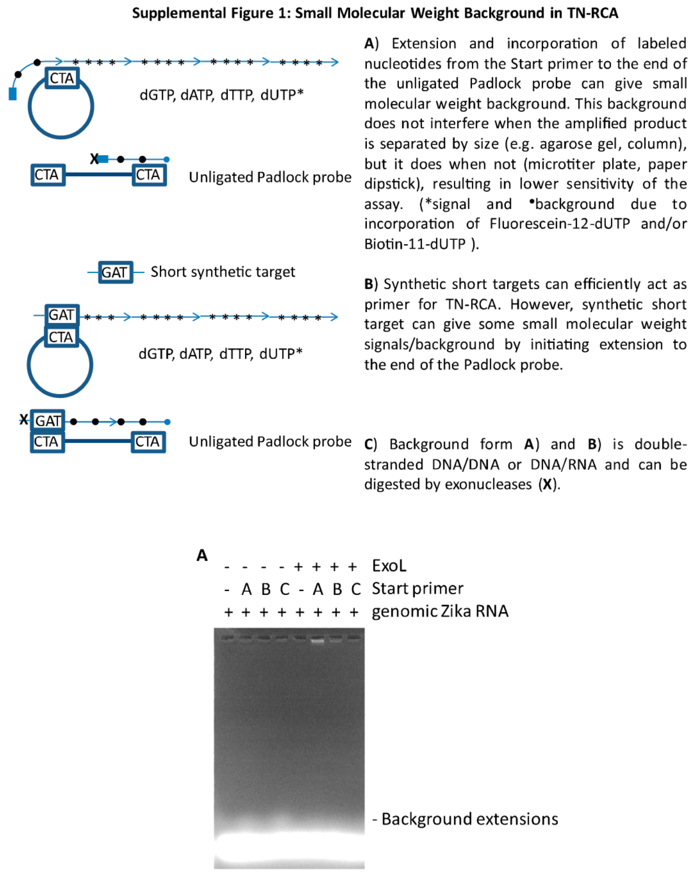

Figure S1. Background reduction of TN-RCA. Scheme of possible origins of background. (**A**) Reduction of small molecular weight background extensions in TN-RCA reaction with genomic Zika RNA originating from three different start primers by Lambda exonuclease (ExoL). Start primers are: A: GfreeStart: 5-TGGTGTTGGTGAGTTAAG-3; B: GfreeStartShort: 5-TGGTGAGTTAAG-3; and C: GfreeStartMismatch: 5-TGTTGTTGTTGAGTTAAG-3. Note that addition of Lambda exonuclease increases TN-RCA amplification efficiency with start primer A (see also Figure S2)

2. Optimization of TN-RCA with exonucleases

TN-RCA in the presence of exonucleases was dependent on the presence of start primer A (**Figure S2**), and since these exonucleases digest from the 5′-end of double-stranded DNA, the most likely explanation is that exonucleases digest the annealed start primer and release torsional strains and steric hindrance arising from extension from the start primer. In fact, when start primers were used that cannot be digested by exonuclease (5′-Biotin, Phosphorothionate) [1], exonuclease did not stimulate amplification, whereas when bona-fide targets of exonuclease were used (5′-Phosphate) [2], increased amplification was observed. In this model, by preventing the formation of a double-stranded circular padlock probe, exonuclease allows free rotation at any of the phosphodiester bond so that the circle can turn 'inside out' erasing any twisting tendency. Free rotation of the circular template may also facilitate slipping of the linear intertwingled genomic target RNA or DNA out of the circular padlock probe and prevent that the newly synthesized strand pulls through the circle once per turn. For short synthetic RNA or DNA target, exonuclease is not required since self-priming extension starts with the target RNA/DNA itself. As described for small circular templates [3,4], unwinding also occurs shortly after synthesis behind the Φ29 polymerase, contrary what is implicated by 'strand-displacement activity' that assumes that the polymerase mainly displaces the already synthesized strand in front of it in the circular padlock probe. Moreover, since Φ29 does not have 5′ to 3′-exonuclease activity, the start primer may interfere in each round of amplification, in particular with larger circular padlock probes and longer start primers with lower torsional strain in the curved circular duplex and higher melting temperature, respectively. In fact, rolling circle replication of large plasmid-sized circles requires unwinding and single-strand binding activities to aid in the displacement of duplex DNA in front of the polymerase [3,4].

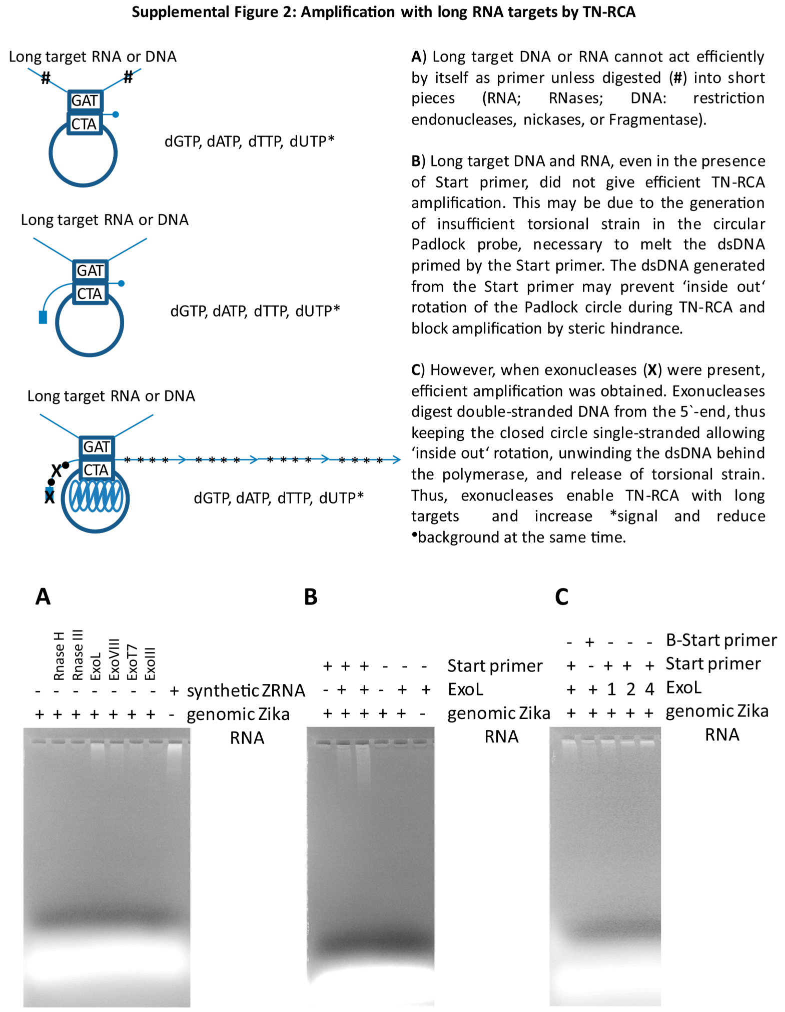

Figure S2. Optimization of TN-RCA with exonucleases. Scheme of possible mechanisms that prevent TN-RCA with genomic targets of DNA or RNA and possible solutions. (**A**) TN-RCA is efficiently increased by additions of exonucleases (lambda, VIII, T7, III), and somewhat by RNase H and shortcut RNase (RNase III). (**B**) Increase of TN-RCA by exonuclease lambda is dependent on the presence of start primer GfreeStart: 5-TGGTGTTGGTGAGTTAAG-3. (**C**) Biotinylated GfreeStart primer (B-Start primer) reduces TN-RCA stimulated by lambda exonuclease (exoL), suggesting involvement of the Start primer in the increase of TN-RCA, increasing the concentration of Lambda exonuclease (1, 2, 4 µL of 0.5 U/µL) only weakly increases TN-RCA.

3. Detection of other DNA and RNA targets as exemplified with human papilloma virus DNA and Noro virus RNA targets

To demonstrate that TN-RCA can be adapted to other target sequences, new padlocks were designed for human papilloma virus (HPV) and the Noro virus.

TN-RCA design for HPV detection

HPV General Primer 6 plus (GP6**+**: 5-GAAAAATAAACTGTAAATCATATTC-3) which allows PCR amplification of 14 high risk HPV subtypes [5] contains only 2 G, and alignment of these HPV virus sequences revealed that the region around GP6+ has a low number of G in all 14 HPV virus variants. Therefore, the following oligonucleotide was used as the 5`-phosphorylated HPV G-free padlock probe (bold: ends that anneal to the extended HPV GP6plus target sequence with 2 G changed to C), and the internal sequence and start primer (underlined) identical to the one used for the Zika virus G-free padlock probe:

G-free padlock for HPV:

5-p-

CATATTCCTCTTTTTATCTTAACTCACCAACACCATTTTTTCTAATCTCAACCTTACTACACT CTTTTTTT**TCAAAAATAAACTCTAAAT-3**

HPV-General target (HPV16, with two mismatches shown lower case):

5-GAGGAATATGATTTAgAGTTTATTTTTgA-3

Start primer:

5-TGGTGTTGGTGAGTTAAG-3

The incorporation of "Universal base analogues" into the padlock sequence opposite the C in the target sequence may help in annealing while still allowing TN-RCA in the presence of only three dNTP [6].

TN-RCA reaction with DNA and G-free padlock probe for human papilloma virus (HPV)

TN-RCA with G-free padlock probe and start primer were essentially performed as for Zika Virus DNA, using either DNA generated by PCR with GP5+ and GP6+ universal primers [5] and with HeLa genomic DNA.

GP5+: 5-TTTGTTACTGTGGTAGATACTAC-3

GP6+: 5-GAAAAATAAACTGTAAATCATATTC-3

Amplification was performed using 2 µL of HeLa genomic DNA (1/1000 diluted from 120 ng/µL stock), 1 µL GP5+ (50 µM (1/20 of 1 mM), 100 pmole/µL), 1 µL GP6+ (50 µM (1/20 of 1 mM), 100 pmole/µL), 6 µL water, 10 µL KOD polymerase (EMD Millipore). Pre-cycling was 2 min denaturation at 95°C, cycling was 10 s denaturation at 95°C, 10 s annealing at 40°C, 10 s extension at 72°C for 40 cycles, and 4 min elongation at 4°C. The PCR products were separated by a 2% agarose gel, extracted with a gel extraction kit (Qiagen) and sequenced (Genewiz) or used for TN-RCA reactions.

TN-RCA reaction with RNA and G-free padlock probe for Noro virus

Norovirus GII sequence was screened for stretches of C-free DNA sequences and candidate sequences checked for uniqueness using NBlast searches, and the following target sequence and padlock probe were selected. TN-RCA was essentially performed as for Zika virus.

Noro G-free padlock 74: (bold: ends that anneal to the extended Noro target sequence, start primer (underlined) identical to the one used for the Zika virus G-free padlock probe:

5-p-

TCATACCAACTCTTTTTAT<u>CTTAACTCACCAACACCA</u>TCTCAACCTTACTACACTCTTTTTT**C**

CTCATTCATAT-3

Noro target sequence (bold):

NORORNA: 5-CUUCCUAUCA**GAGUUGGUAUGAAUAUGAAUGAGG**AUGGCCCCAU-3

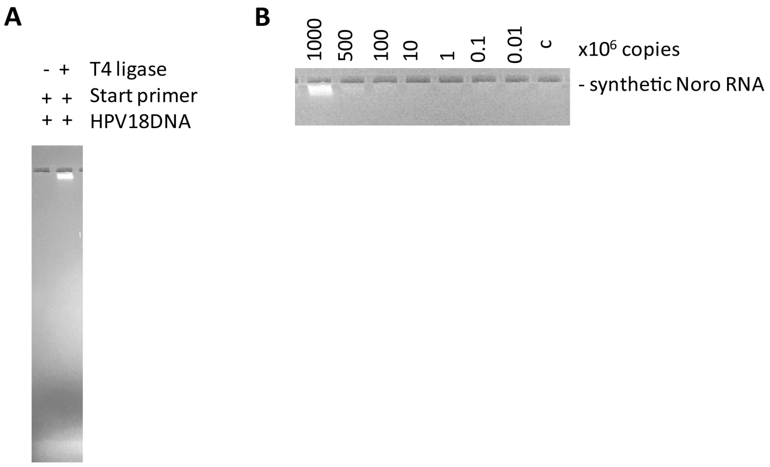

Figure S3. Detection of human papilloma virus (HPV) DNA and Noro virus RNA. (**A**) human papilloma virus 18 (HPV18) DNA amplified from HeLa cells; and (**B**) dilutions of Noro virus synthetic RNA.

4. Development of two-step TN-RCA

Since the basic TN-RCA protocol had a relatively low detection limit, several methods were used to increase it by fragmenting the Fluorescein- and/or Biotin-labeled TN-RCA reaction products after or during the assay. In these experiments, mostly circularized padlock probes (cLPadlocks) were used to evaluate whether the fragments generated in a first TN-RCA amplification can serve as targets and primers for a second TN-RCA amplification. It was found that fragmentation by digestion with a restriction enzyme (MseI) was feasible but required the addition of the complementary G-free oligonucleotide containing the MseI recognition site (**Figure S4A**); the addition of a circularized padlock probe did only weakly increase amplification with the MseI-cut and heat-inactivated first TN-RCA reaction suggesting that the digested fragments did not efficiently serve as targets for a second TN-RCA reaction (**Figure S4B**).

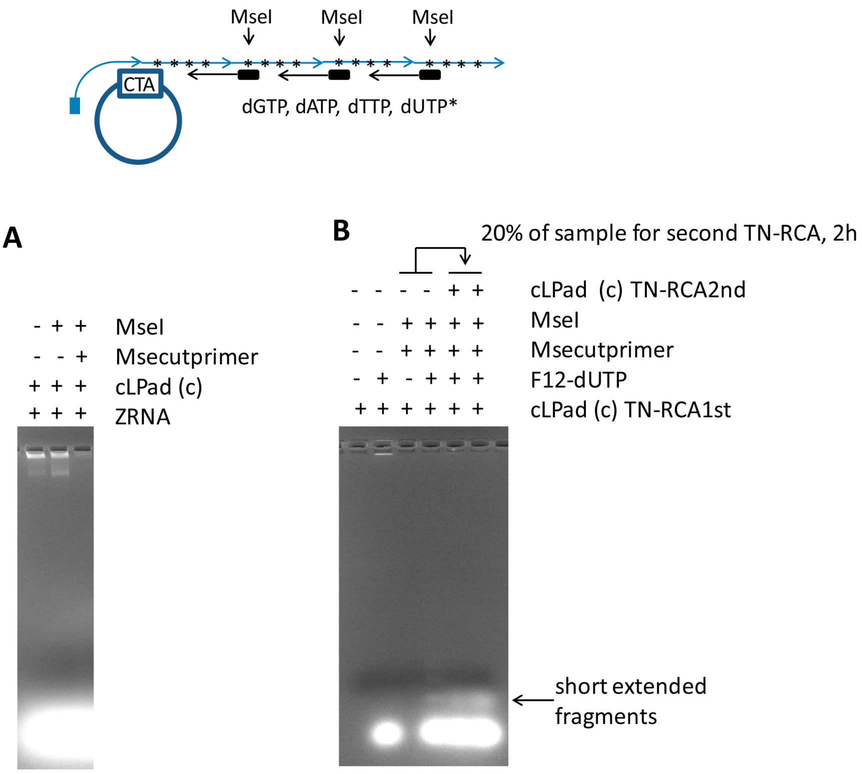

Figure S4. Development of two-step TN-RCA. Scheme of fragmentation of TN-RCA reaction products by MseI. (**A**) TN-RCA was performed with a circularized padlock probe (c) (cLPad (c)) and synthetic ZRNA, and the reaction product cleaved with MseI. Cleavage was only observed when the MseI recognition site was made double-stranded by annealing the complementary oligonucleotide Msecutprimer, 5-TTTATCTTAACTCACCAACT-3 (underlined: MseI recognition site). (**B**) The addition of a circularized padlock probe (cLPad (c) RCA 2nd) to the cleaved TN-RCA reaction products from the first reaction derived from (cLPad (c) RCA 1st) did not increase high molecular weight TN-RCA products, but increased small molecular weight products.

5. Fragmentation of RCA products by Uracil DNA glycosylase and Endonuclease IV

Another method for TN-RCA product fragmentation is by using Uracil DNA glycosylase (UDG) and endonuclease IV which cuts the abasic sites. UDG and endonuclease IV were not able to digest the TN-RCA reaction products with incorporated Fluorescein-12-dUTP or Biotin-11-dUTP (**Figure S5A**). However, UDG alone and in combination of endonuclease IV efficiently fragmented the TN-RCA reaction products containing various amounts of dUTP (**Figure S5B**). However, the presence of endonuclease IV did not enhance much fragmentation over UDG alone. Unexpectedly, in the presence of circularized padlock probes, the presence of endonuclease IV gave strong background signals assumed to be the result of generating too many non-specific starting points for Φ29 [7], so that in subsequent experiments endonuclease IV was not used.

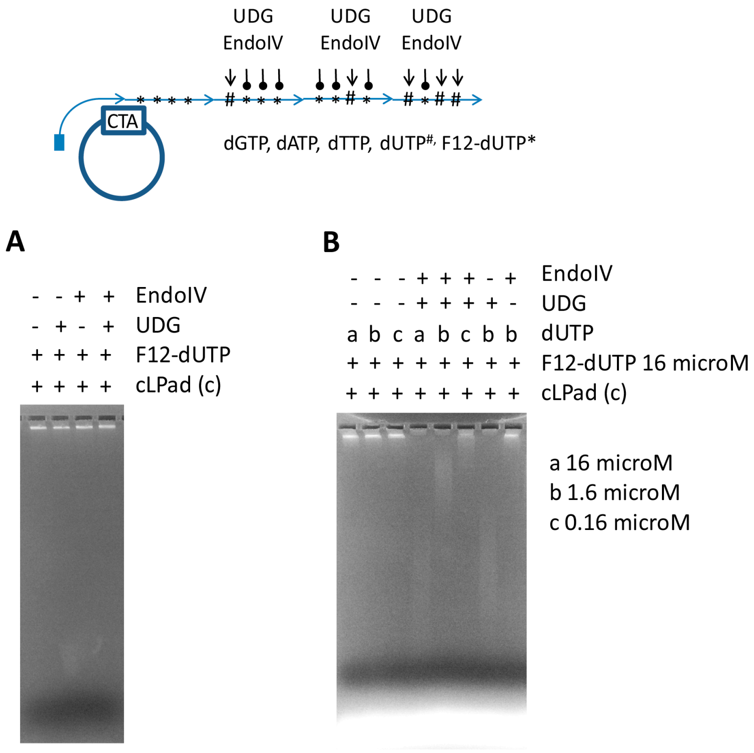

Figure S5. Development of two-step TN-RCA. Scheme of fragmentation of TN-RCA reaction products from ZRNA by Uracil glycosylase (UDG) and endonuclease IV. (**A**) No digestion of TN-RCA reaction products containing Fluorescein-12-dUTP by Uracil glycosylase (UDG) and endonuclease IV (Endo IV). (**B**) Efficient digestion of TN-RCA reaction products containing dUTP (16, 1.6, 0.16 µM) and F12-dUTP (16 µM), but Endonuclease IV did not increase digestion further.

6. Development of two-step protocol with Uracil DNA glycosylase

The UDG fragmented reaction products from the first TN-RCA reaction did not serve as efficient targets for a second complete ligation/amplification TN-RCA reaction, possibly because the ligation reaction is less efficient with targets containing dUTP and abasic sites after UDG digestion (**Figures S6A and S6B**); however, the addition of a circularized padlock probe to the reaction increased TN-RCA amplification with UDG-digested TN-RCA suggesting that the UDG-digested fragments can serve as targets for self-priming (**Figures S6A and S6C**). Secondary TN-RCA amplification was increased by the addition of circularized padlock probes to UDG-digested TN-RCA products from genomic Zika RNA, but no further amplification was observed with products from ZRNA most likely since the signal band was already maximal (**Figures S6D and S6E**), altogether suggesting that the fragments generated in the presence of dUTP and UDG could serve as templates for a secondary TN-RCA reaction.

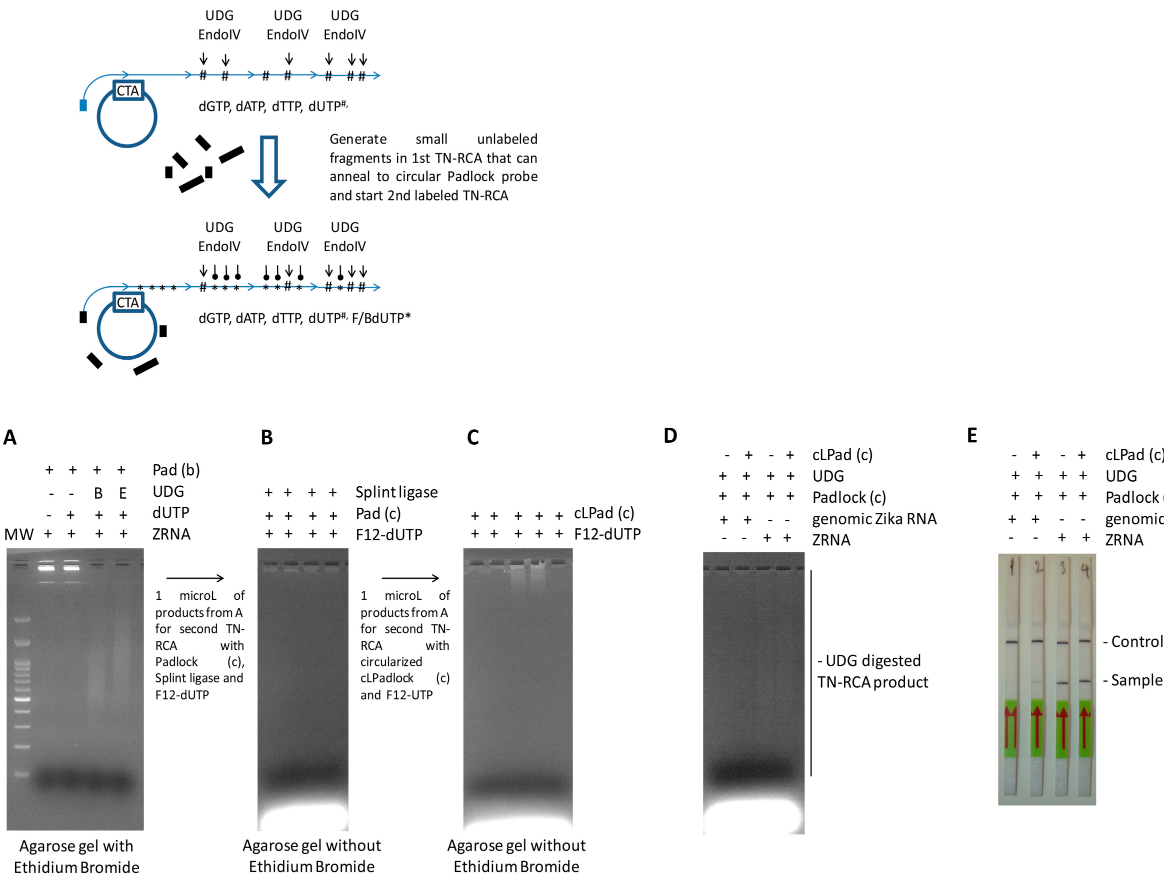

Figure S6. Development of two-step TN-RCA. Scheme of two-step TN-RCA in which fragmented reaction products from a first reaction serve as (self-priming) targets for a second TN-RCA with a circular padlock probe. (**A**) In a first TN-RCA, unlabeled reaction products containing dUTP were produced with padlock probe (b) and synthetic Zika RNA (ZRNA) and digested with UDG (B: UDG added just after the ligation reaction; E: UDG added at the end of the TN-RCA reaction for 15 min). (**B**) The unlabeled TN-RCA reaction products did not serve as efficient targets for a second complete ligation/amplification TN-RCA reaction with F12-dUTP. (**C**) The unlabeled TN-RCA reaction products served as targets for a second TN-RCA reaction with circularized padlock probe (c) (cLPad (c)) and F12-dUTP. (**D**) In the presence of dUTP and UDG, the circular padlock probe (c) (cLPad (c)) stimulates TN-RCA reaction products with genomic Zika RNA and synthetic Zika RNA (ZRNA), but since the UDG-digested fragments are distributed along the entire agarose gel lane they are difficult to see. (**E**) However, the addition of circularized padlock (c) (cLPad (c)) increased amplification as detectable with lateral flow assay on Dipsticks using a 5′-Biotin-labeled Detection probe (1 µL of 50 µM) able to hybridize to the TN-RCA product (Biotin-5-CTCAACCTTACTACACTC-3).

7. Evaluation of influence of background RNA or DNA in TN-RCA (basic protocol)

To determine the influence of background RNA or DNA for TN-RCA and to compare TN-RCA to regular RCA, synthetic target RNA (ZRNA) was spiked into HeLa genomic RNA or DNA either in the presence of only three dNTPs (AGT, TN-RCA) or in the presence of four dNTPs (AGTC, RCA). Although the comparison of amplification conditions with three and four dNTPs using the same G-free padlock can give some idea of the advantages of TN-RCA over RCA, it can be expected that padlocks consisting of four nucleotides as normally used for RCA would increase background even more since at low temperature they may anneal to many different target sequences.

Using the basic protocol (30 min ligation, 2 h amplification), robust amplification was observed with the synthetic Zika RNA (ZRNA). In the absence of ZRNA neither the presence of HeLa genomic RNA (**Figure S7A**), nor the HeLa genomic DNA (**Figure S7B**) gave much background for both TN-RCA and RCA conditions; however, increased background was detected in particular in the presence of four dNTPs (RCA) when spiked into denatured/renatured HeLa genomic DNA, suggesting that sample properties are important for background generation (e. g. accessible 3′-ends may act as start point for the polymerase) (**Figure S7C**).

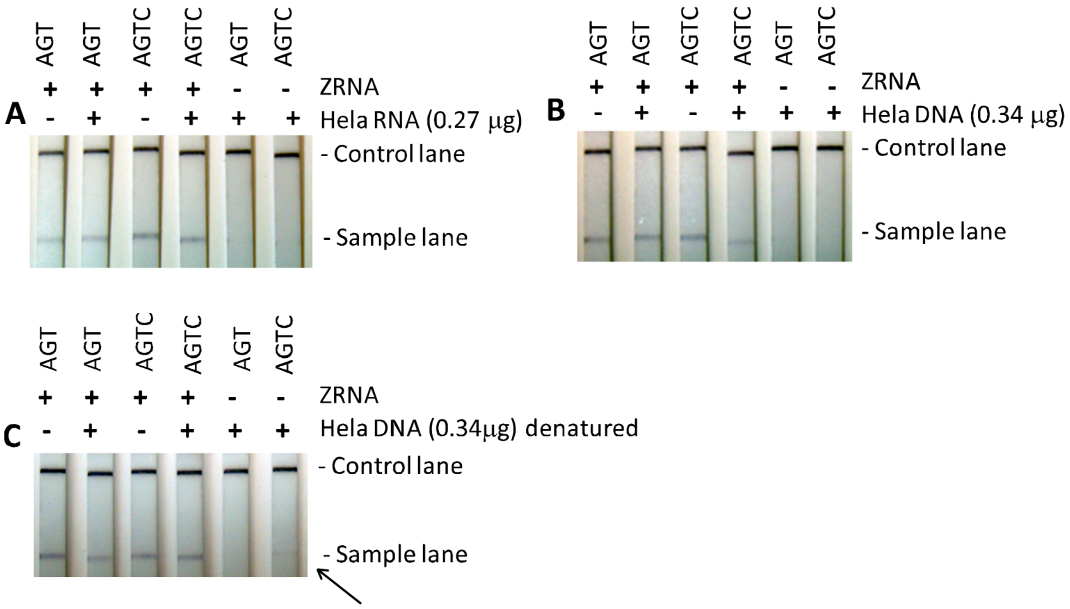

Figure S7. Influence of background RNA or DNA on TN-RCA and RCA with basic protocol. ZRNA ($1x10^7$ copies) was spiked into (**A**) HeLa genomic RNA (0.27 µg), into (**B**) HeLa genomic DNA (0.34 µg), or into (**C**) denatured/renatured HeLa genomic DNA (0.34 µg), and detected in the presence of either three dNTPs (AGT, TN-RCA) or four dNTPs (AGTC, RCA) using the basic protocol as outlined in the methods section. Water spiked into the same HeLa RNA or DNA served as controls. Arrows indicate the presence of background signals.

8. Evaluation of influence of background RNA in TN-RCA (two-step protocol)

A strong background signal was generated when ZRNA was spiked into large amounts of HeLa RNA (2.7 µg) and amplified using the two-step protocol (30 min ligation, 1 h amplification in the presence of dUTP, RNase H, and UDG; 1 h amplification with F12-dUTP and cLPad (c); detection with 5′-Biotin-labeled Detection probe (1 µL of 50 µM)) in the presence of four dNTPs (AGTC, RCA), which was much weaker in the presence of three dNTP (AGT, TN-RCA) (**Figure S8A**). With spiking of ZRNA into 10-fold diluted HeLa RNA (0.27 µg) this background signal almost disappeared (**Figure S8B**). Only a weak signal was detected using these conditions with a genomic Zika RNA sample and a amplification time with the cLPad (c) of 1 h (**Figure S8C**), but lengthening the amplification time with the cLPad (c) to 90 min increased the signal, albeit generated some background with the water control with the TN-RCA conditions and more so with the RCA conditions (**Figure S8D**). These results indicate that for specific applications, the two-step TN-RCA protocol may need to be carefully optimized (purity of sample, time of amplification of each step, amounts of enzymes (e.g. UDG, RNase), amounts and type of circularized padlock, etc) in order to minimize background signal generation.

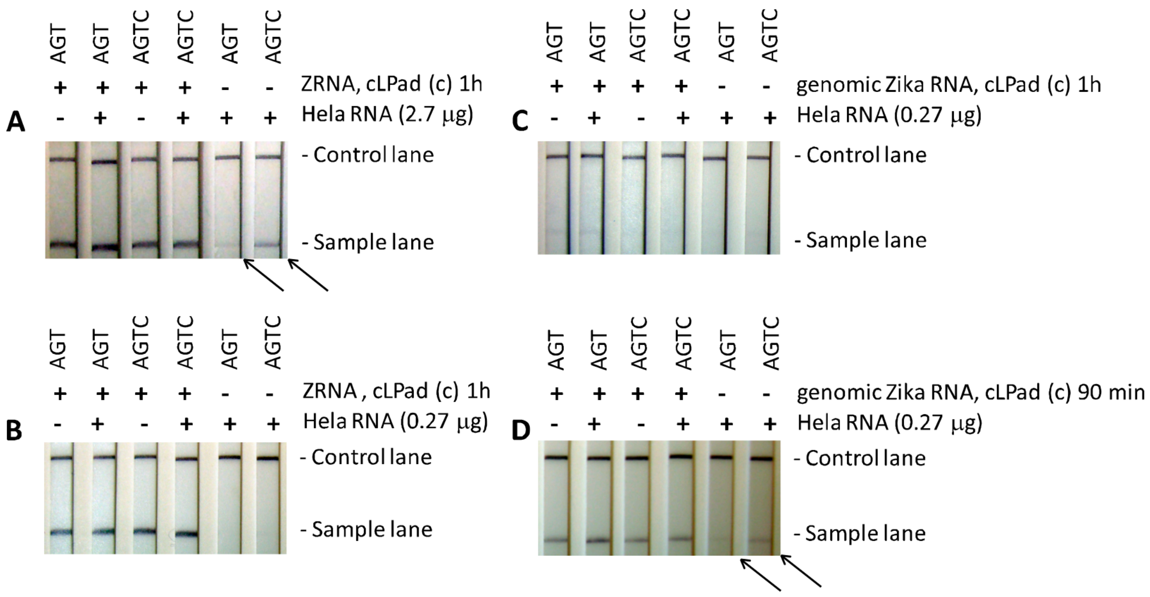

Figure S8. Influence of background RNA on TN-RCA and RCA with two-step protocol. ZRNA ($1x10^7$ copies) was spiked into (A) large amounts of HeLa genomic RNA (2.7 μg), into (**B**) 10 times diluted HeLa genomic RNA (0.27 μg) and detected in the presence of either three dNTPs (AGT, TN-RCA) or four dNTPs (AGTC, RCA) using the two-step protocol as outlined in the methods section and Figure S6. (**C** and **D**) Genomic Zika RNA was spiked into HeLa genomic RNA (0.27 μg) and amplified using the two-step protocol with cLPad (c) for (**C**) 1 h, or for (**D**) 90 min, as outlined in the methods section and in Figure S6. Water spiked into the same HeLa RNA served as controls. Arrows indicate the presence of background signals.

9. Limit of detection in the presence and absence of background RNA with two-step TN-RCA protocol

To assess the influence of background RNA on the limit of detection, synthetic ZRNA was serially diluted and amplified using the two-step TN-RCA protocol in the presence or absence of background HeLa genomic RNA as outlined in the methods section and in Figure S6. In the absence of HeLa genomic RNA, robust amplification was observed until a dilution to 10^5 copies, from where on the signal suddenly dropped (**Figure S9A**). Thus, when compared to the one step basic protocol (**Figure 3**), the two-step protocol appeared to generally strengthen the signal in particular towards low target copy numbers, but did only modestly affect the limit of detection, suggesting that the ligation/initiation step may be a limiting step. A similar pattern appeared when the serially diluted ZRNA was spiked into HeLa genomic RNA; in this case, a higher sample to sample variability was observed with the signal less clearly distinguishable from the background towards the limit of detection (**Figure S9B**).

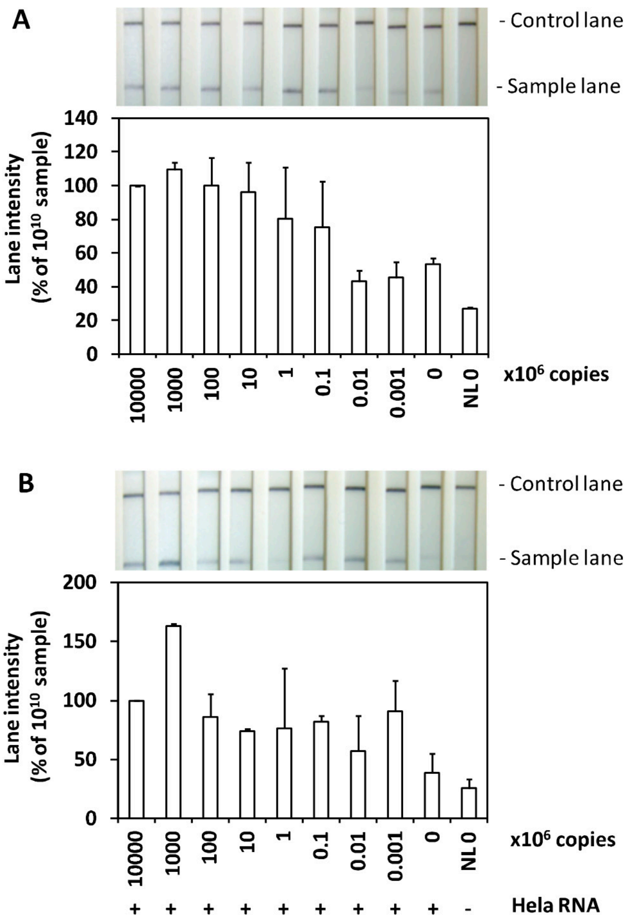

Figure S9. Limit of detection in the presence of background RNA with two step TN-RCA protocol. Serial dilutions of (**A**) ZRNA, and of (**B**) ZRNA spiked into 0.27 µg genomic HeLa RNA, and two-step TN-RCA amplification as outlined in the methods section and in Figures S6 and S8. NL: no-ligase control. Data are expressed in percentage of the sample with 10^{10} copies of ZRNA (n=2). Water spiked into the same HeLa RNA served as controls. Arrows indicate the presence of background signals.

10. Comparison of TN-RCA and RCA

For comparison of the TN-RCA with the RCA method, a 74 bp long RCA padlock was designed that contained the same backbone as the TN-RCA backbone (to minimize additional reasons for background such as self-annealing of padlock and start primer), but at each of the padlock ends 12 bp with all four bases for annealing to a 24 bp Normal Zika target (NDNA), overlapping with 5 bp with the 5′-end of the ZDNA target.

NormalZ padlock 74 for Zika:

5-p-
TTAGCCCGGAACTTTTTATCTTAACTCACCAACACCATCTCAACCTTACTACACTCTTTTTT**CAA CAGCCATCT**-3

Normal Target DNA (NDNA):
5-CCCCACTGTC**GTTCCGGGCTAAAGATGGCTGTTG**GTATGGAATG-3

Start primer:

5-TGGTGTTGGTGAGTTAAG-3

TN-RCA and RCA reactions were run in parallel using the basic protocol (30 min ligation, 2 h amplification), in the presence and absence of HeLa DNA (0.2 μg) as non-specific background. Whereas both methods led to robust amplification in the presence of their DNA targets, the background was generally higher with RCA, in particular in the presence of the HeLa DNA (**Figure S10A**) and more so with denatured HeLa DNA (**Figure S10B**). This can be explained by incorporation of labelled dNTPs into accessible 3′-ends present in denatured HeLa genomic DNA (what is the basis of the whole genome amplification method [8]) and subsequent detection as background by Paper Dipsticks. On agarose gels, HeLa DNA derived background was only visible when Ethidium Bromide was present (**Figure S10C**), as signals co-migrating with the TN-RCA/RCA amplification products as already described in **Figure 2**.

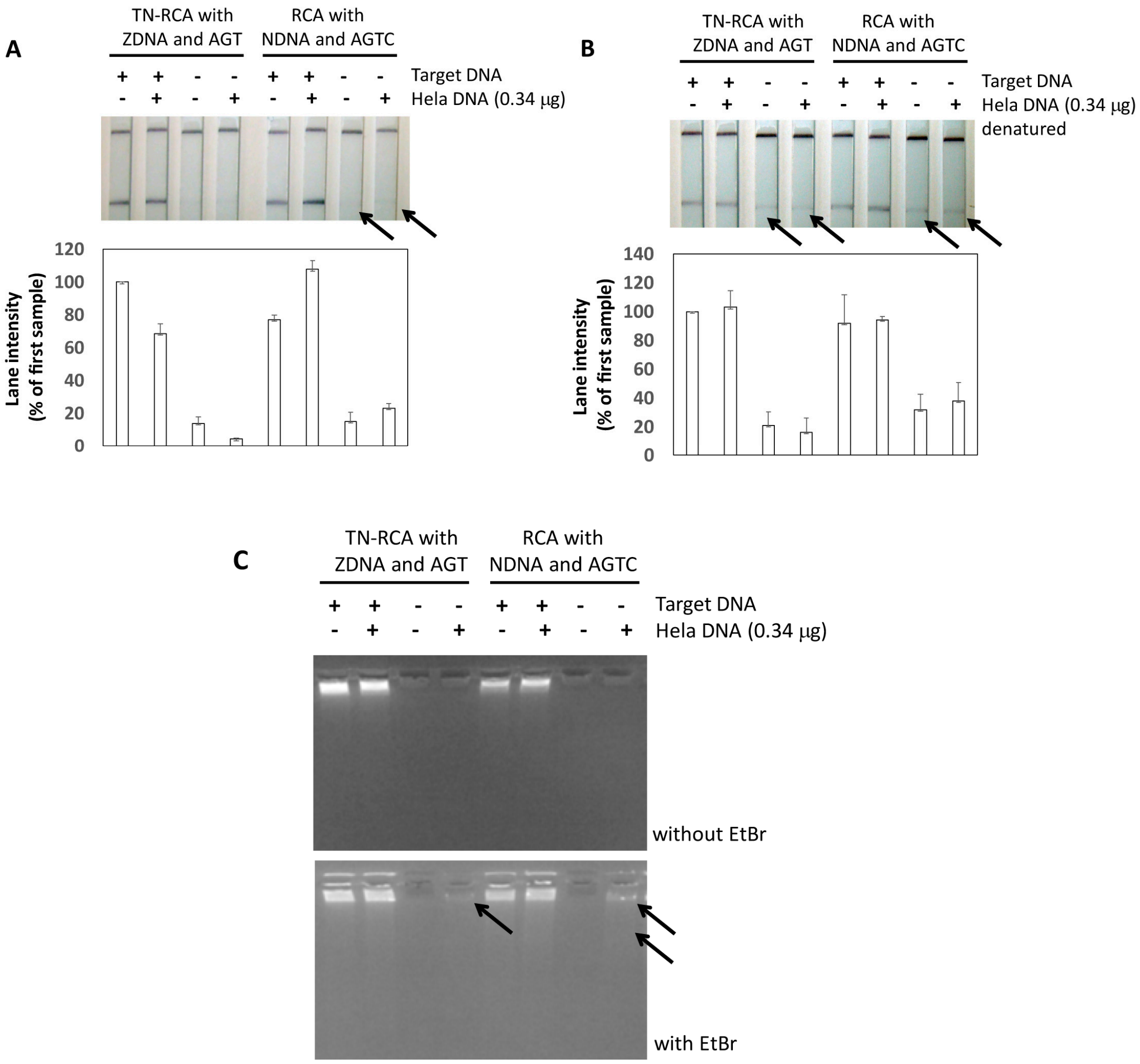

Figure S10. Comparison of methods TN-RCA and RCA in the presence of background DNA. ZDNA and NDNA (10^7 copies) were amplified using TN-RCA (AGT) and RCA (AGCT) conditions, respectively, using the basic protocol as outlined in the methods section. Target DNA was spiked into (**A**) non-denatured and (**B and C**) denatured HeLa DNA (0.34 µg). In (**C**) the agarose gel of (**B**) is shown with and without staining with Ethidium Bromide (EtBr). Data are expressed in percentage of the first sample (n=2). Arrows indicate the presence of background signals.

11. Limit of detection with conventional RCA

To assess the limit of detection with conventional RCA, ZDNA was serially diluted and amplified using basic RCA (AGCT) conditions in the presence background HeLa genomic DNA. Robust amplification was observed until a dilution to 10^7 copies, from where on the signal was difficult to distinguish from background (**Figure S11A**). In contrast, TN-RCA (AGT) gave less background and signals at a dilution of 10^5- 10^6 copies were still distinguishable from background (**Figure S11B**).

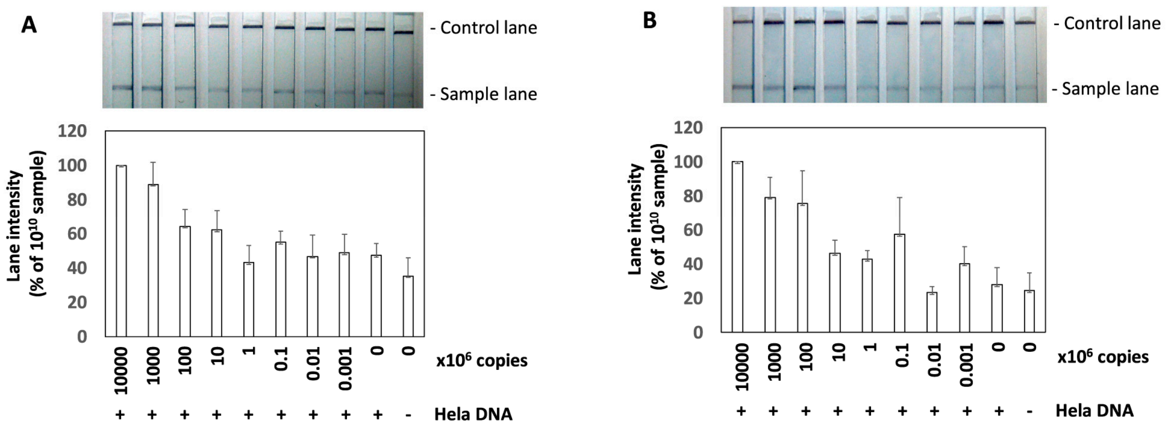

Figure S11. Limit of detection with conventional RCA and TN-RCA using the basic protocol as outlined in the methods section and in Figures S6 and S8. (**A**) Serial dilutions of NDNA into 0.2 µg denatured genomic HeLa DNA and conventional RCA (AGCT). (**B**) Serial dilutions of ZDNA into 0.2 µg denatured genomic HeLa DNA and TN-RCA. Data are expressed in percentage of the sample with 10^{10} copies of NDNA or ZDNA, respectively (n=2).

In conclusion, TN-RCA gave less background than conventional RCA, most likely because conventional RCA requires higher temperatures to avoid mismatched annealing and secondary structure of the padlock/start primer to avoid mismatched ligation and non-specific amplification. However, the performance differences between the TN-RCA and RCA based amplification and detection systems may be influenced by a number of additional factors, such as sample characteristics (amount, composition, single- or double- stranded DNA or RNA, size, purity, degree of degradation), the type of amplification method used (incorporation of one or two labelled dNTPs or use of labelled Detection probe, use of start primer/self priming), and type of method used for detection (agarose gel, microtiter plate, DNA affinity column, lateral flow assay with paper Dipsticks).

REFERENCES

1. Murgha, Y.E.; Rouillard, J.M.; Gulari, E. Methods for the preparation of large quantities of complex single-stranded oligonucleotide libraries. *PLoS One* **2014**, *9*, e94752.

2. Subramanian, K.; Rutvisuttinunt, W.; Scott, W.; Myers, R.S. The enzymatic basis of processivity in lambda exonuclease. *Nucleic acids research* **2003**, *31*, 1585-1596.
3. Fire, A.; Xu, S.Q. Rolling replication of short DNA circles. *Proceedings of the National Academy of Sciences of the United States of America* **1995**, *92*, 4641-4645.
4. Liu, D.; Daubendiek, S.L.; Zillman, M.A.; Ryan, K.; Kool, E.T. Rolling circle DNA synthesis: Small circular oligonucleotides as efficient templates for DNA polymerases. *Journal of the American Chemical Society* **1996**, *118*, 1587-1594.
5. de Roda Husman, A.M.; Walboomers, J.M.; van den Brule, A.J.; Meijer, C.J.; Snijders, P.J. The use of general primers gp5 and gp6 elongated at their 3' ends with adjacent highly conserved sequences improves human papillomavirus detection by pcr. *The Journal of general virology* **1995**, *76 (Pt 4)*, 1057-1062.
6. Loakes, D. Survey and summary: The applications of universal DNA base analogues. *Nucleic acids research* **2001**, *29*, 2437-2447.
7. Salas, M.; Holguera, I.; Redrejo-Rodriguez, M.; de Vega, M. DNA-binding proteins essential for protein-primed bacteriophage phi29 DNA replication. *Frontiers in molecular biosciences* **2016**, *3*, 37.
8. Sujayanont, P.; Chininmanu, K.; Tassaneetrithep, B.; Tangthawornchaikul, N.; Malasit, P.; Suriyaphol, P. Comparison of phi29-based whole genome amplification and whole transcriptome amplification in dengue virus. *Journal of virological methods* **2014**, *195*, 141-147.

© 2018 by the authors. Submitted for possible open access publication under the terms and conditions of the Creative Commons Attribution (CC BY) license (http://creativecommons.org/licenses/by/4.0/).
